# Synthesis and Biological Evaluation of Novel Phosphatidylcholine Analogues Containing Monoterpene Acids as Potent Antiproliferative Agents

**DOI:** 10.1371/journal.pone.0157278

**Published:** 2016-06-16

**Authors:** Anna Gliszczyńska, Natalia Niezgoda, Witold Gładkowski, Marta Czarnecka, Marta Świtalska, Joanna Wietrzyk

**Affiliations:** 1 Department of Chemistry, Wrocław University of Environmental and Life Sciences, Norwida 25, 50–375 Wrocław, Poland; 2 Ludwik Hirszfeld Institute of Immunology and Experimental Therapy, Polish Academy of Sciences, Department of Experimental Oncology, Weigla 12, 53–114 Wrocław, Poland; Louisiana State University Health Sciences Center, UNITED STATES

## Abstract

The synthesis of novel phosphatidylcholines with geranic and citronellic acids in *sn*-1 and *sn*-2 positions is described. The structured phospholipids were obtained in high yields (59–87%) and evaluated *in vitro* for their cytotoxic activity against several cancer cell lines of different origin: MV4-11, A-549, MCF-7, LOVO, LOVO/DX, HepG2 and also towards non-cancer cell line BALB/3T3 (normal mice fibroblasts). The phosphatidylcholines modified with monoterpene acid showed a significantly higher antiproliferative activity than free monoterpene acids. The highest activity was observed for the terpene-phospholipids containing the isoprenoid acids in *sn*-1 position of phosphatidylcholine and palmitic acid in *sn*-2.

## Introduction

Isoprenoids are one of the most widely occurring group of natural compounds. Many of them exhibit wide range of biological activities. Among documented useful properties of isoprenoids the following activities need to be mentioned: fungicidal, antibacterial, antiviral and anti-inflammatory [1–4]. The results of many studies show that they inhibit proliferation of cancer cell lines, causing inhibition of cell division and transition of cells into the phase of programmed cell death [5,6]. The administered doses, even in high concentrations do not cause such an effect in the case of healthy cells [7].

In recent years there has been also a rapid progress in the field of bioengineering and phospholipid technology [8,9]. Many new discoveries in this area completely changed the idea that phospholipids are just the integral components of biomembrane ensuring their integrity and functionality and energetic and supplementary material [10,11]. It is currently known that phospholipids are also involved in many metabolic and neurological reactions [12,13]. They regulate fundamental biological processes and play an important role in the transmission of information as intracellular mediators [14,15]. For the description of phospholipids-protein interactions, the mechanisms of their action in membrane as signaling molecules and relation between their structure and activity the key point is preparation of a great number of phospholipid derivatives. Especially important in this area is development of new synthetic methods for the preparation of biologically active phospholipids (PLs) with a defined structure and high purity.

Keeping in view of the properties of isoprenoids and phospholipids the production of novel hybrid molecules with the combination of these two biologically important products could result in bioactive analogues—terpene-phospholipids, which could be characterized by enhanced biological activities. It has been reported that phospholipid modifications of drugs is the method of modulation their polarity, to prevent degradation and enhance the biological activity of molecules attached to the PLs [16–20].

## Materials and Methods

### General Procedures

Geranic acid (mixture of *cis*/t*rans* isomers; 23:67) (**1a**) and racemic citronellic acid (**1b**) were purchased from Sigma–Aldrich Chemical Co. Phospholipase A_2_ (Lecitase 10L; 10,000 LEU/mL) was a gift from Novozymes. The enantiomerically pure GPC was purchased from Bachem and converted to the cadmium chloride complex (GPC **×** CdCl_2_) using the method described elsewhere. All of the solvents used in liquid chromatography were of HPLC grade (LiChrosolv Reagents) and were purchased from Merck.

Analytical TLC was performed on silica gel 60, F_254_ plates (Merck) with mixture of chloroform: methanol: water (65:25:4; v/v/v) as developing system. Products were detected by spraying the plates with a solution of 10 g of Ce(SO_4_)_2_ and 20 g of phosphomolybdic acid in 1 L of 10% H_2_SO_4_ followed by heating or 0.05% primuline solution (8: 2; acetone/H2O; v/v) followed by UV (365 nm). Column chromatography was performed on silica gel (Kieselgel 60, 230–400 mesh (Merck)). ^1^H, ^13^C, and ^31^P NMR spectra were recorded using a Bruker Advance II 600 MHz spectrometer. Chemical shifts are given in ppm downfield from tetramethylsilane (TMS) as the internal standard. In ^31^P NMR spectra, chemical shifts were referenced to 85% H_3_PO_4_ as a standard. Coupling constant (*J*) values are given in hertz. Elemental analyses were performed on Waters ESI-Q-TOF Premier XE spectrometer using electron spray ionization (ESI) technique.

The purity of synthesized products were monitored by HPLC using an Ultimate 3000 DIONEX chromatograph equipped with a DGP-3600A dual-pump fluid control module, a TCC-3200 thermostatted column compartment, and a WPS-3000 autosampler. A Corona charged aerosol detector (CAD) from ESA Biosciences was used. The acquisition range 100 pA, digital filter set to none, the N_2_ pressure was 0.24 MPa. The system was controlled and data acquisition was carried out using the *Chromeleon 6*.*80* software (Dionex Corporation). HPLC analysis was carried out using a Betasil DIOL 5-μm column (Thermo, 150 × 2.1 mm).

The column temperature was maintained at 30°C. The gradient had a constant flow rate 1.5 mL min^-1^ with solvent A = 1% HCOOH, 0.1% triethylamine (TEA) in water, B = hexane, and C = propan-2-ol. Gradient time: 3/40/57 (%A/%B/%C), at 4 min 10/40/50, at 9 min 10/40/50, at 9.1 min 3/40/57 and at 19 min 43/40/57. The total time of analysis was 19 min. The injection volume was 15 μL in all of the experiments and the cooling temperature for the samples was 20°C.

### Preparation of Cadmium Chloride Complex of GPC (GPC × CdCl_2_)

The solution of *sn*-glycero-3-phosphocholine (GPC) (10 g, 38.7 mmol) in methanol (65 mL) was added to a solution of cadmium chloride (CdCl_2_ × 2^1^/_2_H_2_O) (8.88 g, 38.7 mmol) in cold water (30 mL). A heavy white precipitate appeared and was stored at 0°C for 4 h. The precipitate (GPC × CdCl_2_) was next filtered, lyophilized and then dried in an Abderhalden’s drying pistol over P_2_O_5_ at the boiling temperature of acetone to give 15.65 g of white powder in 91% yield.

### General procedure for the 1,2-diacylation of GPC × CdCl_2_ (3a, 3b)

The cadmium complex of GPC has been acetylated with isoprenoid acids in positions *sn*-1 and *sn*-2. Geranic acid (**1a**) or citronellic acid (**1b**) (0.92 mmol) was dried by repeated evaporation with an anhydrous CH_2_Cl_2_ and then dissolved in anhydrous CH_2_Cl_2_ (1 mL). To the 100 mg (0.23 mmol) of GPC × CdCl_2_ complex added 56 mg (0.46 mmol) of DMAP and the solution of 200 mg of DCC (0.97 mmol) in 6 mL of the anhydrous CH_2_Cl_2_. The reaction flask was flushed with dry N_2_ and next tightly sealed. The mixture was stirred at room temperature under protection from light for 72 h. After this time (TLC) the formed during the reaction course precipitate was removed using a Schott funnel. The solution was mixed with ion-exchange resin (DOWEX 50W X8, H^+^ form) for 30 min to dislodge the cadmium chloride and DMAP. DOWEX was filtered off and the solvent was evaporated under vacuum. The crude PC was purified by silica gel column chromatography (65: 25: 4, CHCl_3_/CH_3_OH/H_2_O, (v/v/v)). Fractions containing products (*R*_f_ 0.3) were collected and evaporated under vacuum (45°C) to afford 1,2-diisoprenoyl-*sn*-glycero-3-phosphocholine **3** as a mixture of two stereoisomers.

#### 1',2'-di(*E*)-geranoyl)-*sn*-glycero-3'-phosphocholine + 1',2'-di(*Z*)-geranoyl)-*sn*-glycero-3'-phosphocholine (3a)

Colourless greasy solid (82% yield, 99% purity (according to HPLC)); ^**1**^**H NMR** (600 MHz, CDCl_3_/CD_3_OD 2:1 (v/v)), δ: 1.29 (two s, 12H, CH_3_-9_sn-1_ (A), CH_3_-9_sn-1_ (B), CH_3_-9_sn-2_ (A), CH_3_-9_sn-2_ (B)), 1.36 (s, 12H, CH_3_-8_sn-1_ (A), CH_3_-8_sn-1_ (B), CH_3_-10_sn-2_ (A), CH_3_-10_sn-2_ (B)), 1.81–1.85 (m, 8H, CH_2_-4_sn-1_ (A), CH_2_-4_sn-1_ (B), CH_2_-5_sn-2_ (A), CH_2_-5_sn-2_ (B)), 2.91 (s, 18H, -N(CH_3_)_3_ (A), N(CH_3_)_3_ (B)), 3.32–3.35 (m, 4H, CH_2_-β (A), CH_2_-β (B)), 3.70–3.77 (m, 4H, CH_2_-3' (A), CH_2_-3' (B)), 3.91 (dd, *J* = 12.0, 6.6 Hz, 2H, one of CH_2_-1' (A), one of CH_2_-1' (B)), 3.96–4.01 (m, 4H, CH_2_-α (A), CH_2_-α (B), 4.06 (m, 2H, one of CH_2_-1' (A), one of CH_2_-1' (B), 4.80 (m, 2H, H-6_sn-1_ (B), H-6_sn-2_ (B)), 4.84 (m, 2H, H-6_sn-1_ (A), H-6_sn-2_ (A)), 4.94 (m, 2H, H-2' (A), H-2' (B)), 5.35 (s, 4H, H-2_sn-1_ (A), H-2_sn-1_ (B), H-2_sn-2_ (A), H-2_sn-2_ (B)); ^**13**^**C NMR** (151 MHz, CDCl_3_/CD_3_OD 2:1 (v/v)) δ: 15.39, 15.44, 15.46, 15.49, 24.70, 24.74, 24.88 (C-8_sn-1_ (A), C-8_sn-1_ (B), C-8_sn-2_ (A), C-8_sn-2_ (B), C-10_sn-1_ (A), C-10_sn-1_ (B), C-10_sn-2_ (A), C-10_sn-2_ (B)), 16.88, 16.89, 16.93, 16.95 (C-9_sn-1_ (A), C-9_sn-1_ (B), C-9_sn-2_ (A), C-9_sn-2_ (B)), 25.58, 25.59, 26.26, 26.28 (C-5_sn-1_ (A), C-5_sn-1_ (B), C-5_sn-2_ (A), C-5_sn-2_ (B)), 40.52, 40.54, 40.55, 40.57 (C-4_sn-1_ (A), C-4_sn-1_ (B), C-4_sn-2_ (A), C-4_sn-2_ (B)), 53.65 (t, *J* = 3.7 Hz, -N(**C**H_3_)_3_ (A), -N(**C**H_3_)_3_ (B)), 58.59 (d, *J* = 4.8 Hz, C-α (A), C-α (B)), 61.73 (C-1' (A), C-1' (B)), 63.24 (d, *J* = 4.8 Hz, C-3' (A), C-3' (B)), 66.10 (m, C-β (A), C-β (B)), 70.28 (d, *J* = 8.4 Hz, C-2' (A), C-2' (B)), 114.49, 114.60, 115.00, 115.10 (C-2_sn-1_ (A), C-2_sn-1_ (B), C-2_sn-2_ (A), C-2_sn-2_ (B)), 121.85, 121.88, 122.35, 122.37 (C-6_sn-1_ (A), C-6_sn-1_ (B), C-6_sn-2_ (A), C-6_sn-2_ (B)), 128.11, 128.15 (C-7_sn-1_ (A), C-7_sn-1_ (B), C-7_sn-2_ (A), C-7_sn-2_ (B)), 131.44, 132.13 (C-3_sn-1_ (A), C-3_sn-1_ (B), C-3_sn-2_ (A), C-3_sn-2_ (B)), 161.05, 161.19, 165.69, 165.78 (C-1_sn-1_ (A), C-1_sn-1_ (B), C-1_sn-2_ (A), C-1_sn-2_ (B)); ^**31**^**P NMR** (243 MHz, CDCl_3_/CD_3_OD 2:1 (v/v)) δ: -0.63; HRMS (ESI): *m/z* calcd. for C_28_H_48_NO_8_P [M + H]^+^ 558.3196; found 558.3217

#### (2'*R*)-1',2'-di[(3*R*)-3,7-dimethylocta-6-enyl]-*sn*-glycero-3'-phosphocholine + (2'*R*)-1',2'-di[(3*S*)-3,7-dimethylocta-6-enyl]-*sn*-glycero-3'-phosphocholine (3b)

Colourless greasy solid (86% yield, 99% purity (according to HPLC)); ^**1**^**H NMR** (600 MHz, CDCl_3_/CD_3_OD 2:1 (v/v)), δ: 0.70, 0.71 (two d, *J* = 7.8 Hz, 12H, CH_3_-9_sn-1_ (A), CH_3_-9_sn-1_ (B), CH_3_-9_sn-2_ (A), CH_3_-9_sn-2_ (B)), 0.99 (m, 4H, one of CH_2_-4_sn-1_ (A), one of CH_2_-4_sn-1_ (B), one of CH_2_-4_sn-2_ (A), one of CH_2_-4_sn-2_ (B)), 1.12 (m, 4H, one of CH_2_-4_sn-1_ (A), one of CH_2_-4_sn-1_ (B), one of CH_2_-4_sn-2_ (A), one of CH_2_-4_sn-2_ (B)), 1.36, 1.44 (two s, 24H, CH_3_-8_sn-1_ (A), CH_3_-8_sn-1_ (B), CH_3_-10_sn-1_ (A), CH_3_-10_sn-1_ (B), CH_3_-8_sn-2_ (A), CH_3_-8_sn-2_ (B), CH_3_-10_sn-2_ (A), CH_3_-10_sn-2_ (B)), 1.66–1.81 (m, 12H, CH_2_-5_sn-1_ (A), CH_2_-5_sn-1_ (B), CH_2_-5_sn-2_ (A), CH_2_-5_sn-2_ (B), H-3_sn-1_ (A), H-3_sn-1_ (B), H-3_sn-2_ (A), H-3_sn-2_ (B)), 1.86–1.95 (m, 4H, one of CH_2_-2_sn-1_ (A), one of CH_2_-2_sn-1_ (B), one of CH_2_-2_sn-2_ (A), one of CH_2_-2_sn-2_ (B)), 2.07–2.14 (m, 4H, one of CH_2_-2_sn-1_ (A), one of CH_2_-2_sn-1_ (B), one of CH_2_-2_sn-2_ (A), one of CH_2_-2_sn-2_ (B)), 2.99 (s, 18H, -N(CH_3_)_3_ (A), N(CH_3_)_3_ (B)), 3.38–3.39 (m, 4H, CH_2_-β (A), CH_2_-β (B)), 3.77 (m, 4H, CH_2_-3' (A), CH_2_-3' (B)), 3.92 (m, 2H, one of CH_2_-1' (A), one of CH_2_-1' (B)), 4.03 (m, 4H, CH_2_-α (A), CH_2_-α (B)), 4.19 (m, 2H, one of CH_2_-1' (A), one of CH_2_-1' (B)), 4.84 (t, *J* = 7.2 Hz, 4H, H-6_sn-1_ (A), H-6_sn-1_ (B), H-6_sn-2_ (A), H-6_sn-2_ (B)), 5.00 (m, 2H, H-2' (A), H-2' (B)); ^**13**^**C NMR** (151 MHz, CDCl_3_/CD_3_OD 2:1 (v/v)) δ: 16.91, 24.96 (C-8_sn-1_ (A), C-8_sn-1_ (B), C-8_sn-2_ (A), C-8_sn-2_ (B), C-10_sn-1_ (A), C-10_sn-1_ (B), C-10_sn-2_ (A), C-10_sn-2_ (B)), 18.77, 18.78. 18.85 (C-9_sn-1_ (A), C-9_sn-1_ (B), C-9_sn-2_ (A), C-9_sn-2_ (B)), 24.88 (C-5_sn-1_ (A), C-5_sn-1_ (B), C-5_sn-2_ (A), C-5_sn-2_ (B)), 29.49, 29.53, 29.56 (C-3_sn-1_ (A), C-3_sn-1_ (B), C-3_sn-2_ (A), C-3_sn-2_ (B)), 36.20, 36.23 (C-4_sn-1_ (A), C-4_sn-1_ (B), C-4_sn-2_ (A), C-4_sn-2_ (B)), 40.99, 41.15, 41.16 (C-2_sn-1_ (A), C-2_sn-1_ (B), C-2_sn-2_ (A), C-2_sn-2_ (B)), 53.58 (t, *J* = 3.5 Hz, -N(**C**H_3_)_3_ (A), -N(**C**H_3_)_3_ (A)), 58.75 (d, *J* = 4.8 Hz, C-α (A), C-α (B)), 62.21 (C-1' (A), C-1' (B)), 63.27 (d, *J* = 4.7 Hz, C-3' (A), C-3' (B)), 65.93 (m, C-β (A), C-β (B)), 69.89, (d, *J* = 7.7 Hz, C-2' (A)), 69.91 (d, *J* = 8.3 Hz, C-2' (B)), 123.64 (C-6_sn-1_ (A), C-6_sn-1_ (B), C-6_sn-2_ (A), C-6_sn-2_ (B)), 131.09 (C-7_sn-1_ (A), C-7_sn-1_ (B), C-7_sn-2_ (A), C-7_sn-2_ (B)), 172.55, 172.56, 172.97 (C-1_sn-1_ (A), C-1_sn-1_ (B), C-1_sn-2_ (A), C-1_sn-2_ (B)); ^**31**^**P NMR** (243 MHz, CDCl_3_/CD_3_OD 2:1 (v/v)) δ: -0.79; HRMS (ESI): *m/z* calcd. for C_28_H_52_NO_8_P [M + H]^+^ 562.3509; found 562.3530

### General procedure for 1,2-dipalmitoyl-*sn*-glycero-3-phosphocholine enzymatic hydrolysis

Hydrolysis of 1,2-dipalmitoyl-*sn*-glycero-3-phosphocholine (**5**) was carried out according to the method described by Morgado et al. [21]. The detailed data were described earlier [22]. 1-Palmitoyl-2-hydroxy-*sn*-glycero-3-phosphocholine **(6)** was obtained in 99% yield and its physical and spectroscopic data were consistent with those found in literature [23].

### General procedure for 1-palmitoyl-2-isoprenoyl-*sn*-glycero-3-phosphocholine (7a, 7b) synthesis

Terpene acids (**1a** or **1b**, 0.604 mmol), solution of DMAP (74 mg, 0.6 mmol) in 3 mL of CH_2_Cl_2_ and finally a solution of DCC (267 mg, 1.3 mmol) in 5 mL of CH_2_Cl_2_ were added to the solution of 1-palmitoyl-2-hydroxy-*sn*-3-glycero-phosphocholine **(6)** (150 mg, 0.302 mmol) dissolved in 4 mL of anhydrous CH_2_Cl_2_. The reaction was carried out for 72 h under a nitrogen atmosphere in the dark at 40°C. The product was extracted according to the method described for compound **3a** and **3b**. 1-Palmitoyl-2-isoprenoyl-*sn*-glycero-3-phosphocholine (**7**) was obtained as the mixture of two stereoisomers.

#### 1'-palmitoyl-2'-(*E*)-geranoyl-*sn*-glycero-3'-phosphocholine + 1'-palmitoyl-2'-(*Z*)-geranoyl-*sn*-glycero-3'-phosphocholine (7a)

Colourless greasy solid (59% yield, 98% purity (according to HPLC)); ^**1**^**H NMR** (600 MHz, CDCl_3_/CD_3_OD 2:1 (v/v)) δ: 0.60 (t, *J* = 6.6 Hz, 6H, C**H**_**3**_(CH_2_)_14_C(O) (A), C**H**_**3**_(CH_2_)_14_C(O) (B)), 1.98–1.02 (m, 48H, CH_3_(C**H**_**2**_)_**12**_CH_2_CH_2_C(O) (A), CH_3_(C**H**_**2**_)_**12**_CH_2_CH_2_C(O) (B)), 1.32 (m, 4H, CH_3_(CH_2_)_13_C**H**_**2**_CH_2_C(O) (A), CH_3_(CH_2_)_13_C**H**_**2**_CH_2_C(O) (B)), 1.35 (s, 6H, CH_3_-9 (A), CH_3_-9 (B)), 1.41 (s, 12H, CH_3_-8 (A), CH_3_-8 (B), CH_3_-10 (A), CH_3_-10 (B)), 1.85–1.91 (m, 8H, CH_2_-4 (A), CH_2_-4 (B), CH_2_-5 (A), CH_2_-5 (B)), 2.03 (m, 4H, CH_3_(CH_2_)_13_C**H**_**2**_C(O) (A), CH_3_(CH_2_)_13_C**H**_**2**_C(O) (B)), 2.94 (s, 18H, -N(CH_3_)_3_ (A), -N(CH_3_)_3_ (B)), 3.31–3.35 (m, 4H, CH_2_-β (A), CH_2_-β (B)), 3.72 (m, 4H, CH_2_-3' (A), CH_2_-3' (B)), 3.92 (m, 2H, one of CH_2_-1' (A), one of CH_2_-1' (B), 3.96–4.00 (m, 4H, CH_2_-α (A), CH_2_-α (B)), 4.14 (m, 2H, one of CH_2_-1' (A), one of CH_2_-1' (B)), 4.80, 4.85 (two t, *J* = 6.6 Hz, 2H, H-6 (A), H-6 (B)), 4.96–5.05 (two m, 2H, H-2' (A), H-2' (B)), 5.39 (s, 2H, H-2 (A), H-2 (B)); ^**13**^**C NMR** (151 MHz, CDCl_3_/CD_3_OD 2:1 (v/v)) δ: 13.37 (**C**H_3_CH_2_(CH_2_)_11_CH_2_CH_2_C(O) (A), **C**H_3_CH_2_(CH_2_)_11_CH_2_CH_2_C(O) (B)), 16.94, 16.95 (C-9 (A), C-9 (B)), 22.15 (CH_3_**C**H_2_(CH_2_)_11_CH_2_CH_2_C(O) (A), CH_3_**C**H_2_(CH_2_)_11_CH_2_CH_2_C(O) (B)), 24.33, 24.34 (CH_3_CH_2_(CH_2_)_11_**C**H_2_CH_2_C(O) (A), CH_3_CH_2_(CH_2_)_11_**C**H_2_CH_2_C(O) (B)), 24.90 (C-8 (A), C-10 (A), C-8 (B), C-10 (B)), 25.53, 25.55 (C-5(A), C-5 (B)), 28.63, 28.77, 28.84, 28.98, 29.11, 29.13, 29.16 (CH_3_CH_2_(**C**H_2_)_11_CH_2_CH_2_C(O) (A), CH_3_CH_2_(**C**H_2_)_11_CH_2_CH_2_C(O) (B), 33.54 (CH_3_CH_2_(CH_2_)_11_CH_2_**C**H_2_C(O) (A), CH_3_CH_2_(CH_2_)_11_CH_2_**C**H_2_C(O) (B)), 40.53, 40.56 (C-4 (A), C-4 (B)), 53.56 (m, -N(**C**H_3_)_3_ (A), -N(**C**H_3_)_3_ (B)), 58.59 (d, *J* = 4.7 Hz, C-α (A), C-α (B)), 62.20 (C-1' (A), C-1' (B)), 63.06, 63.11 (two d, *J* = 5.2 Hz, C-3' (A), C-3' (B)), 65.95–65.99 (two m, C-β (A), C-β (B)), 70.24, 70.31 (two d, *J* = 7.8 Hz, C-2' (A), C-2' (B)), 114.93, 114.41 (C-2 (A), C-2 (B)), 121.78, 121.80 (C-6 (A), C-6 (B)), 126.89, 127.14 (C-7 (A), C-7 (B)), 127.70, 128.12 (C-3 (A), C-3 (B)), 161.45, 165.68 (C-1 (A), C-1 (B)), 173.57, 173.68 (CH_3_CH_2_(CH_2_)_11_CH_2_CH_2_**C**(O) (A), CH_3_CH_2_(CH_2_)_11_CH_2_CH_2_**C**(O) (B)); ^**31**^**P NMR** (243 MHz, CDCl_3_/CD_3_OD 2:1 (v/v)) δ: 0.64; HRMS (ESI): *m/z* calcd. for C_34_H_64_NO_8_P [M + H]^+^ 646.4448; found 646.4451

#### (2'*R*)-1'-palmitoyl-2'-[(3*R*)-3,7-dimethylocta-6-enyl]-*sn*-glycero-3'-phosphocholine + (2'*R*)-1'-palmitoyl-2'-[(3*S*)-3,7-dimethylocta-6-enyl]-*sn*-glycero-3'-phosphocholine (7b)

Colourless greasy solid (62% yield, 99% purity (according to HPLC)); ^**1**^**H NMR** (600 MHz, CDCl_3_/CD_3_OD 2:1 (v/v)), δ: 0.64 (t, *J* = 6.6 Hz, 6H, C**H**_**3**_(CH_2_)_14_C(O) (A), C**H**_**3**_(CH_2_)_14_C(O) (B)), 0.72 (d, *J* = 7.2 Hz, 6H, CH_3_-9 (A), CH_3_-9 (B)), 0.96–1.05 (m, 50H, CH_3_(C**H**_**2**_)_**12**_CH_2_CH_2_C(O) (A), CH_3_(C**H**_**2**_)_**12**_CH_2_CH_2_C(O) (B), one of CH_2_-4 (A), one of CH_2_-4 (B)), 1.12 (m, 2H, one of CH_2_-4 (A), one of CH_2_-4 (B)), 1.31–1.39 (m, 4H, CH_3_(CH_2_)_13_C**H**_**2**_CH_2_C(O) (A), CH_3_(CH_2_)_13_C**H**_**2**_CH_2_C(O) (B)), 1.36, 1.44 (two s, 12H, CH_3_-8 (A), CH_3_-8 (B), CH_3_-10 (A), CH_3_-10 (B)), 1.65–1.81 (m, 6H, CH_2_-5 (A), CH_2_-5 (B), H-3 (A), H-3 (B)), 1.87–1.93 (two dd, *J* = 15.0, 8.4 Hz, 2H, one of CH_2_-2 (A), one of CH_2_-2 (B)), 2.07 (t, *J* = 7.8 Hz, 4H, CH_3_(CH_2_)_13_C**H**_**2**_C(O) (A), CH_3_(CH_2_)_13_C**H**_**2**_C(O) (B)), 2.09–2.15 (two dd, *J* = 15.0, 6.0 Hz, 1H, one of CH_2_-2 (A), one of CH_2_-2 (B)), 2.99 (s, 18H, -N(**C**H_3_)_3_ (A), -N(**C**H_3_)_3_ (B)), 3.39 (m, 4H, CH_2_-β (A), CH_2_-β (B)), 3.77 (m, 4H, CH_2_-3' (A), CH_2_-3’ (B), 3.92 (m, 2H, one of CH_2_-1' (A), one of CH_2_-1' (B)), 4.03 (m, 4H, CH_2_-α (A), CH_2_-α (B)), 4.17 (m, 2H, one of CH_2_-1' (A), one of CH_2_-1' (B)), 4.84 (t, *J* = 6.6 Hz, 2H, H-6 (A), H-6 (B)), 5.00 (m, 2H, H-2' (A), H-2' (B)); ^**13**^**C NMR** (151 MHz, CDCl_3_/CD_3_OD 2:1 (v/v)) δ: 13.44 (**C**H_3_(CH_2_)_14_C(O) (A), **C**H_3_(CH_2_)_14_C(O) (B)), 16.97, 25.02 (C-8 (A), C-8 (B), C-10 (A), C-10 (B)), 18.93 (C-9 (A), C-9 (B)), 22.20 (CH_3_**C**H_2_(CH_2_)_13_C(O) (A), CH_3_**C**H_2_(CH_2_)_13_C(O) (B)), 24.45 (CH_3_(CH_2_)_12_**C**H_2_CH_2_C(O) (A), (CH_3_(CH_2_)_12_**C**H_2_CH_2_C(O) (B)), 24.94 (C-5 (A), C-5 (B)), 28.67, 28.87, 28.89, 29.06, 29.19, 29.21, 29.23, 31.47 (CH_3_CH_2_(**C**H_2_)_11_CH_2_CH_2_C(O) (A), CH_3_CH_2_(**C**H_2_)_11_CH_2_CH_2_C(O) (B)), 29.55, 29.56 (C-3 (A), C-3 (B)), 33.78 (CH_3_(CH_2_)_13_**C**H_2_C(O) (A), CH_3_(CH_2_)_13_**C**H_2_C(O) (B)), 36.28 (C-4 (A), C-4 (B)), 41.06 (C-2 (A), C-2 (B)), 53.66 (t, *J* = 3.6 Hz, -N(**C**H_3_)_3_ (A), -N(**C**H_3_)_3_ (B)), 58.79 (d, *J* = 4.9 Hz, C-α (A), C-α (B)), 62.12, 62.14 (C-1' (A), C-1' (B)), 63.39 (d, *J* = 5.1 Hz, C-3' (A), C-3' (B)), 65.00 (m, C-β (A), C-β (B)), 69.95 (d, *J* = 7.8 Hz, C-2' (A), C-2' (B)), 123.68 (C-6 (A), C-6 (B)), 131.13 (C-7 (A), C-7 (B)), 172.95 (C-1 (A), C-1 (B)), 173.19 (CH_3_(CH_2_)_13_CH_2_**C**(O) (A), CH_3_(CH_2_)_13_CH_2_**C**(O) (B); ^**31**^**P NMR** (243 MHz, CDCl_3_/CD_3_OD 2:1 (v/v)) δ: -0.83; HRMS (ESI): *m/z* calcd. for C_34_H_66_NO_8_P [M + H]^+^ 648.4604; found 648.4615

### General procedure the synthesis of 1-isoprenoyl-2-hydroxy-*sn*-glycero-3-phosphocholine (9a, 9b)

The synthesis of 1-isoprenoyl-2-hydroxy-*sn*-glycero-3-phosphocholine (**9a**, **9b**) was carried out according to the procedure described by Niezgoda et al. [24]. GPC (300 mg, 1.16 mmol) and dibutyltin oxide (DBTO) (290 mg, 1.17 mmol) were suspended in 10 mL of anhydrous propan-2-ol and refluxed for 1 h. The reaction was cooled to an ambient temperature and TEA (2.4 mmol) was added followed by terpene chloride (281 mg, 2.4 mmol). After 1 h of the reaction, the mixture was filtreted using diatomaceous earth (Celite^®^ 545) and washed CH_2_Cl_2_. The organic solvent was evaporated under vacuum (45°C), after which the crude residue was purified on a silica gel column (65: 25: 4 CHCl_3_/CH_3_OH/H_2_O, (v/v/v)). Product-containing fractions of *R*_f_ 0.1 were collected and solvent evaporated under vacuum (45°C) to gave 1-isoprenoyl-2-hydroxy-*sn*-glycero-3-phosphocholine **(9a, 9b)**. The chemical structure was confirmed by ^1^H and ^13^C NMR spectra.

#### 1'-(*E*)-geranoyl-2'-hydroxy-*sn*-glycero-3'-phosphocholine + 1'-(*Z*)-geranoyl-2'-hydroxy-*sn*-glycero-3'-phosphocholine) (9a)

Colourless greasy solid (75% yield, 99% purity (according to HPLC)); ^**1**^**H NMR** (600 MHz, CDCl_3_/CD_3_OD 2:1 (v/v)), δ: 1.38 (s, 6H, CH_3_-9 (A), CH_3_-9 (B)), 1.45 (s, 24H, CH_3_-8 (A), CH_3_-8 (B), CH_3_-10 (A), CH_3_-10 (B)), 1.89–1.84 (m, 8H, CH_2_-4 (A), CH_2_-4 (B), CH_2_-5 (A), CH_2_-5 (B)), 2.99 (s, 18H, -N(CH_3_)_3_ (A), -N(CH_3_)_3_ (B)), 3.37–3.39 (m, 4H, CH_2_-β (A), CH_2_-β (B)), 3.65 (m, 4H, one of CH_2_-3' (A), one of CH_2_-3' (B)), 3.71–3.78 (two m, 4H, H-2' (A), H-2' (B), one of CH_2_-3' (A), one of CH_2_-3' (B)), 3.87–3.96 (two m, 4H, CH_2_-1' (A), CH_2_-1' (B)), 4.02–4.05 (m, 4H, CH_2_-α (A), CH_2_-α (B)), 4.86 (m, 2H, H-6 (A), H-6 (B)), 5.48 (s, 2H, H-2 (A), H-2 (B)); ^**13**^**C NMR** (151 MHz, CDCl_3_/CD_3_OD 2:1 (v/v)) δ: 17.00, 18.24 (C-9 (A), C-9 (B)), 24.97 (C-8 (A), C-8 (B), C-10 (A), C-10 (B)), 25.58 (C-5 (A), C-5 (B)), 40.56, 41.19 (C-4 (A), C-4 (B)), 53.63 (t, *J* = 3.8 Hz, -N(**C**H_3_)_3_ (A), -N(**C**H_3_)_3_ (B)), 58.63 (d, *J* = 4.9 Hz, C-α (A), C-α (B)), 64.88 (C-1' (A), C-1' (B)), 66.03 (m, C-β (A), C-β (B)), 66.41 (d, *J* = 5.6 Hz, C-3' (A)), 66.58 (d, *J* = 5.7 Hz, C-3' (B)), 68.24 (d, *J* = 6.6 Hz, C-2' (A)), 68.41 (d, *J* = 6.8 Hz, C-2' (B)), 113.22, 114.58 (C-2 (A), C-2 (B)), 122.41, 123.12 (C-6 (A), C-6 (B)), 131.51, 132.13 (C-7 (A), C-7 (B)), 141.68 (C-3 (A), C-3 (B)), 160.88, 166.63 (C-1 (A), C-1 (B)); ^**31**^**P NMR** (243 MHz, CDCl_3_/CD_3_OD 2:1 (v/v)) δ: 0.02; HRMS (ESI): *m/z* calcd. for C_18_H_34_NO_7_P [M + H]^+^ 408.2151; found 408.2175

#### (2'*R*)-1'-[(3*R*)-3,7-dimethylocta-6-enyl]-2'-hydroxy-*sn*-glycero-3'-phosphocholine + (2'*R*)-1'-[(3*S*)-3,7-dimethylocta-6-enyl]-2'-hydroxy-*sn*-glycero-3'-phosphocholine (9b)

Colourless greasy solid (87% yield, 98% purity (according to HPLC)); ^**1**^**H NMR** (600 MHz, CDCl_3_/CD_3_OD 2:1 (v/v)), δ: 0.72 (d, *J* = 6.6 Hz, 6H, CH_3_-9 (A), CH_3_-9 (B)), 0.96–1.15 (two m, 4H, CH_2_-4 (A), CH_2_-4 (B)), 1.37, 1.45 (two s, 12H, CH_3_-8 (A), CH_3_-8 (B), CH_3_-10 (A), CH_3_-10 (B)), 1.70–1.82 (m, 6H, CH_2_-5 (A), CH_2_-5 (B), H-3 (A), H-3 (B)), 1.93 (dd, *J* = 15.0, 8.4 Hz, 2H, one of CH_2_-2 (A), one of CH_2_-2 (B)), 2.14 (dd, *J* = 15.0, 5.4 Hz, 2H, one of CH_2_-2 (A), one of CH_2_-2 (B)), 2.99 (s, 18H, -N(CH_3_)_3_ (A), -N(CH_3_)_3_ (B)), 3.37–3.39 (m, 4H, CH_2_-β (A), CH_2_-β (B)), 3.62–3.73 (two m, 4H, CH_2_-3' (A), CH_2_-3' (B)), 3.74–3.77 (m, 2H, H-2' (A), H-2' (B)), 3.87–3.95 (two m, 4H, CH_2_-1' (A), CH_2_-1' (B)), 4.02–4.04 (m, 4H, CH_2_-α (A), CH_2_-α (B)), 4.85 (t, *J* = 7.2 Hz, 2H, H-6 (A), H-6 (B)); ^**13**^**C NMR** (151 MHz, CDCl_3_/CD_3_OD 2:1 (v/v)) δ: 16.92, 24.98 (C-8 (A), C-8 (B) C-10 (A), C-10 (B)), 18.86 (C-9 (A), C-9 (B)), 24.91 (C-5 (A), C-5 (B)), 29.53 (C-3 (A), C-3 (B)), 36.30 (C-4 (A), C-4 (B)), 41.09 (C-2 (A), C-2 (B)), 53.61 (t, *J* = 3.8 Hz, -N(**C**H_3_)_3_ (A), -N(**C**H_3_)_3_ (B), 58.62 (d, *J* = 5.1 Hz, C-α (A), C-α (B)), 64.49 (C-1' (A), C-1' (B)), 66.01 (m, C-β (A), C-β (B)), 66.47 (d, *J* = 5.6 Hz, C-3' (A), C-3' (B)), 68.30 (d, *J* = 6.5 Hz, C-2' (A), C-2' (B)), 123.72 (C-6 (A), C-6 (B)), 131.08 (C-7 (A), C-7 (B)), 173.39 (C-1 (A), C-1 (B)); ^**31**^**P NMR** (243 MHz, CDCl_3_/CD_3_OD 2:1 (v/v)) δ: -0.03; HRMS (ESI): *m/z* calcd. for C_18_H_36_NO_7_P [M + H]^+^ 410.23075; found 410.2311

### General procedure for the synthesis of 1-isoprenoyl-2-palmitoyl-*sn*-glycero-3-phosphocholine (10a, 10b)

Compounds **10a** and **10b** were prepared from 1-isoprenoyl-2-hydroxy-*sn*-glycero-3-phosphocholines (**9a, 9b**) (0.14 mmol) by adding palmitic acid (0.56 mmol) dissolved in 2 mL of anhydrous CH_2_Cl_2_, DMAP (0.28 mmol) and finally a solution of DCC (0.58 mmol) in 3 mL of CH_2_Cl_2_. The reaction was carried out for 72 h under a nitrogen atmosphere in the dark at 40°C. The product was extracted according to the method described for compounds **3a, 3b** and pure 1-isoprenoyl-2-palmitoyl-*sn*-glycero-3-phosphocholine (**10**) was obtained as the mixture of two stereoisomers.

#### 1'-(*E*)-geranoyl-2'-palmitoyl-*sn*-glycero-3'-phosphocholine + 1'-(*Z*)-geranoyl-2'-palmitoyl-*sn*-glycero-3'-phosphocholine) (10a)

Colourless greasy solid (67% yield, 99% purity (according to HPLC)); ^**1**^**H NMR** (600 MHz, CDCl_3_/CD_3_OD 2:1 (v/v)) δ: 0.63 (t, *J* = 6.6 Hz, 6H, C**H**_**3**_(CH_2_)_14_C(O) (A), C**H**_**3**_(CH_2_)_14_C(O) (B)), 1.03–1.04 (m, 48H, CH_3_(C**H**_**2**_)_**12**_CH_2_CH_2_C(O)), 1.35 (m, 4H, CH_3_(CH_2_)_13_C**H**_**2**_CH_2_C(O) (A), CH_3_(CH_2_)_13_C**H**_**2**_CH_2_C(O) (B)), 1.36 (s, 6H, CH_3_-9 (A), CH_3_-9 (B)), 1.43 (s, 12H, CH_3_-8, CH_3_-10), 1.86–1.92 (two m, 8H, CH_2_-4 (A), CH_2_-4 (B), CH_2_-5 (A), CH_2_-5 (B)), 2.07 (t, *J* = 6.6 Hz, 4H, CH_3_(CH_2_)_13_C**H**_**2**_C(O) (A), CH_3_(CH_2_)_13_C**H**_**2**_C(O) (B)), 2.98 (s, 18H, -N(CH_3_)_3_ (A), -N(CH_3_)_3_ (B),), 3.36–3.39 (m, 4H, CH_2_-β (A), CH_2_-β (B)), 3.77 (m, 4H, CH_2_-3' (A), CH_2_-3' (B)), 3.94 (m, 2H, one of CH_2_-1' (A), one of CH_2_-1' (B)), 4.00–4.05 (m, 4H, CH_2_-α (A), CH_2_-α (B)), 4.18 (m, 2H, one of CH_2_-1' (A), one of CH_2_-1' (B)), 4.83 (m, 2H, H-6 (A). H-6 (B)), 4.96–5.03 (m, 2H, H-2' (A), H-2' (B)), 5.41 (s, 2H, H-2 (A), H-2 (B)); ^**13**^**C NMR** (151 MHz, CDCl_3_/CD_3_OD 2:1 (v/v)) δ: 13.38 (**C**H_3_CH_2_(CH_2_)_11_CH_2_CH_2_C(O) (A), **C**H_3_CH_2_(CH_2_)_11_CH_2_CH_2_C(O) (B)), 16.95, 16.97 (C-9 (A), C-9 (B)), 22.16 (CH_3_**C**H_2_(CH_2_)_11_CH_2_CH_2_C(O) (A), CH_3_**C**H_2_(CH_2_)_11_CH_2_CH_2_C(O) (B)), 24.38, 24.43 (CH_3_CH_2_(CH_2_)_11_**C**H_2_CH_2_C(O) (A), CH_3_CH_2_(CH_2_)_11_**C**H_2_CH_2_C(O) (B)), 24.92 (C-8 (A), C-8 (B), C-10 (A), C-10 (B)), 25.55 (C-5 (A), C-5 (B)), 28.59, 28.63, 28.82, 28.85, 29.01, 29.14, 29.16, 29.18 (CH_3_CH_2_(**C**H_2_)_11_CH_2_CH_2_C(O) (A), CH_3_CH_2_(**C**H_2_)_11_CH_2_CH_2_C(O) (B)), 33.70, 33.76 (CH_3_(CH_2_)_13_**C**H_2_C(O) (A), CH_3_(CH_2_)_13_**C**H_2_C(O) (B)), 40.54, 41.13 (C-4 (A), C-4 (B)), 53.57 (t, *J* = 3.2 Hz, -N(**C**H_3_)_3_ (A), -N(**C**H_3_)_3_ (A)), 58.69 (d, *J* = 4.7 Hz, C-α (A), C-α (B)), 62.50 (C-1' (A), C-1' (B)), 63.17 (d, *J* = 5.1 Hz, C-3' (A)), 63.37 (d, *J* = 4.8 Hz, C-3' (B)), 65.94 (m, C-β (A), C-β (B)), 69.85 (d, *J* = 7.8 Hz, C-2' (A)), 70.08 (d, *J* = 8.0 Hz, C-2' (B)), 113.31, 114.32 (C-2 (A), C-2 (B)), 122.32, 123.03 (C-6 (A), C-6 (B)), 131.48, 132.09 (C-7 (A), C-7 (B)), 141.46 (C-3 (A), C-3 (B)), 161.13, 166.16 (C-1 (A), C-1 (B)), 173.13, 173.23 (CH_3_CH_2_(CH_2_)_11_CH_2_CH_2_**C**(O) (A), CH_3_CH_2_(CH_2_)_11_CH_2_CH_2_**C**(O) (B)); ^**31**^**P NMR** (243 MHz, CDCl_3_/CD_3_OD 2:1 (v/v)) δ: 0.83; HRMS (ESI): *m/z* calcd. for C_34_H_64_NO_8_P [M + H]^+^ 646.4448; found 646.4474

#### (2'*R*)-2'-palmitoyl-1'-[(3*R*)-3,7-dimethylocta-6-enyl]-*sn*-glycero-3'-phosphocholine + (2'*R*)-2'-palmitoyl-1'-[(3*S*)-3,7-dimethylocta-6-enyl]-*sn*-glycero-3'-phosphocholine (10b)

Colourless greasy solid (70% yield, 99% purity (according to HPLC)); ^**1**^**H NMR** (600 MHz, CDCl_3_/CD_3_OD 2:1 (v/v)) δ: 0.64 (t, *J* = 6.6 Hz, 6H, C**H**_**3**_(CH_2_)_14_C(O) (A), C**H**_**3**_(CH_2_)_14_C(O) (B)), 0.70 (d, *J* = 6.6 Hz, 6H, CH_3_-9 (A), CH_3_-9 (B)), 0.90–1.34 (m, 50H, CH_3_(C**H**_**2**_)_**12**_CH_2_CH_2_C(O) (A), CH_3_(C**H**_**2**_)_**12**_CH_2_CH_2_C(O) (B), one of CH_2_-4 (A), one of CH_2_-4 (B)), 1.13 (m, 2H, one of CH_2_-4 (A), one of CH_2_-4 (B)), 1.35–1.40 (m, 4H, CH_3_(CH_2_)_13_C**H**_**2**_CH_2_C(O) (A), CH_3_(CH_2_)_13_C**H**_**2**_CH_2_C(O) (B)), 1.36, 1.44 (two s, 12H, CH_3_-8 (A), CH_3_-8 (B), CH_3_-10 (A), CH_3_-10 (B)), 1.68–1.78 (m, 6H, CH_2_-5 (A), CH_2_-5 (B), H-3 (A), H-3 (B)), 1.87–1.92 (m, 2H, one of CH_2_-2 (A), one of CH_2_-2 (B)), 2.06–2.12 (m, 2H, one of CH_2_-2 (A), one of CH_2_-2 (B)), 2.09 (t, *J* = 6.6 Hz, 4H, CH_3_(CH_2_)_13_C**H**_**2**_C(O) (A), CH_3_(CH_2_)_13_C**H**_**2**_C(O) (B)), 2.99 (s, 18H, -N(**C**H_3_)_3_ (A), -N(**C**H_3_)_3_ (B)), 3.39–3.40 (m, 4H, CH_2_-β (A), CH_2_-β (B)), 3.78 (m, 4H, CH_2_-3' (A), CH_2_-3’ (B), 3.93 (m, 2H, one of CH_2_-1' (A), one of CH_2_-1' (B)), 4.01–4.04 (m, 4H, CH_2_-α (A), CH_2_-α (B)), 4.20 (m, 2H, one of CH_2_-1' (A), one of CH_2_-1' (B)), 4.84 (t, *J* = 6.0 Hz, 2H, H-6 (A), H-6 (B)), 4.99 (m, 2H, H-2' (A), H-2' (B)); ^**13**^**C NMR** (151 MHz, CDCl_3_/CD_3_OD 2:1 (v/v)) δ: 13.61 (**C**H_3_(CH_2_)_14_C(O) (A), **C**H_3_(CH_2_)_14_C(O) (B)), 17.14, 25.19 (C-8 (A), C-8 (B), C-10 (A), C-10 (B)), 18.94, 18.97 (C-9 (A), C-9 (B)), 22.31 (CH_3_**C**H_2_(CH_2_)_13_C(O) (A), CH_3_**C**H_2_(CH_2_)_13_C(O) (B)), 24.49 (CH_3_(CH_2_)_12_**C**H_2_CH_2_C(O) (A), (CH_3_(CH_2_)_12_**C**H_2_CH_2_C(O) (B)), 25.03 (C-5 (A), C-5 (B)), 28.78, 28.94, 28.99, 29.14, 29.27, 29.29, 29.30, 29.32, 31.57 (CH_3_CH_2_(**C**H_2_)_11_CH_2_CH_2_C(O) (A), CH_3_CH_2_(**C**H_2_)_11_CH_2_CH_2_C(O) (B)), 29.69, 29.71 (C-3 (A), C-3 (B)), 33.72 (CH_3_(CH_2_)_13_**C**H_2_C(O) (A), CH_3_(CH_2_)_13_**C**H_2_C(O) (B)), 36.34, 36.36 (C-4 (A), C-4 (B)), 41.30 (C-2 (A), C-2 (B)), 53.81 (t, *J* = 3.5 Hz, -N(**C**H_3_)_3_ (A), -N(**C**H_3_)_3_ (B)), 58.86 (d, *J* = 4.8 Hz, C-α (A), C-α (B)), 62.41, 62.43 (C-1' (A), C-1' (B)), 63.42 (d, *J* = 4.8 Hz, C-3' (A), C-3' (B)), 66.09 (m, C-β (A), C-β (B)), 69.95 (d, *J* = 7.8 Hz, C-2' (A)), 69.96 (d, *J* = 7.7 Hz, C-2' (B)), 123.76 (C-6 (A), C-6 (B)), 131.27 (C-7 (A), C-7 (B)), 172.66, 172.68 (C-1 (A), C-1 (B)), 173.71 (CH_3_(CH_2_)_13_CH_2_**C**(O) (A), CH_3_(CH_2_)_13_CH_2_**C**(O) (B)); ^**31**^**P NMR** (243 MHz, CDCl_3_/CD_3_OD 2:1 (v/v)) δ: -0.88; HRMS (ESI): *m/z* calcd. for C_34_H_66_NO_8_P [M + H]^+^ 648.4604; found 648.4630

### Biological studies

#### Cell cultures

Established *in vitro*, human cancer cell lines: MV4-11 (human biphenotypic B myelomonocytic leukaemia), A-549 (non-small cell lung cancer), MCF-7 (breast cancer), LoVo (colon cancer), LoVo/DX (colon cancer drug resistant), HepG2 (liver cancer) and BALB/3T3 (normal mice fibroblasts) were used.

All lines were obtained from American Type Culture Collection (Rockville, Maryland, U.S.A.). The cell line is being maintained in the Institute of Immunology and Experimental Therapy, Wroclaw, Poland.

The leukaemia cell line was cultured in RPMI 1640 medium (IIET, Wroclaw, Poland) with supplemented with 2 mM L-glutamine adjusted to contain 1.0 mM sodium pyruvate, 10% fetal bovine serum (all from Sigma-Aldrich Chemie GmbH, Steinheim, Germany). A549, LoVo and LoVo/DX cells were cultured in the mixture of RPMI 1640+Opti-MEM (1:1) (both from Gibco, Scotland, UK), MCF-7 cells in Eagle medium (IIET, Wroclaw, Poland), HepG2 in DMEM medium (Gibco, Scotland, UK), BALB/3T3 in DMEM medium (IIET, Wroclaw, Poland) supplemented with 2 mM L-glutamine and 1.0 mM sodium pyruvate, 10% fetal bovine serum (all from Sigma-Aldrich, Germany). The MCF-7 cell culture was supplemented with 0.8 mg/L of insulin (Sigma-Aldrich Chemie GmbH, Steinheim, Germany) and the LoVo/DX cell culture was supplemented with 0.1 μg/mL of doxorubicin (Accord). All culture media were supplemented with 100 units/mL penicillin, and 100 μg/mL streptomycin (both from Polfa Tarchomin S.A., Warsaw, Poland). All cell lines were grown at 37°C with 5% CO_2_ humidified atmosphere.

#### Compounds

Prior to usage, the compounds were dissolved in DMSO or in mixture of 99.8% ethanol and DMSO (1:1) to the concentration of 25 or 50 mM, and subsequently diluted in culture medium to reach the required concentrations (ranging from 5 to 625 μM).

#### Antiproliferative assay *in vitro*

24 hours before addition of the tested compounds, the cells were plated in 96-well plates at density of 1×10^4^ cells per well. An assay was performed after 72 hours exposure to varying concentrations of the tested agents.

Cytotoxic test MTT was applied for the cytotoxicity screening against leukaemia cells growing in suspension culture and were carried out according to the method described before [25]. Cytotoxic test SRB was applied against A549, MCF-7, LoVo, LoVo/DX, BALB/3T3 and HepG2 cells and were carried out according to the method described before [25].

The results were calculated as an IC_50_ (inhibitory concentration 50)–the concentration of tested agent, which inhibits proliferation of 50% of the cell population. IC_50_ values were calculated for each experiment separately and mean values ± SD is presented in the Table 1. Each compound in each concentration was tested in triplicate in a single experiment, which was repeated 3–5 times.

**Table 1:** Antiproliferative activity of terpene-phospholipids against selected cell lines.

Compounds	Acyl residue		Cell line /IC_50_ ±SD (μM)							
	*sn*-1	*sn*-2	MV4-11	A-549	MCF-7	LOVO	LOVO/DX	HepG2	BALB/3T3
**1a** geranic acid	-	-	310.77 ± 96.41	n.a.	447.09 ± 72.02	263.1 ± 32	492.3 ± 44.5	n.a.	n.a.	
**1b** citronellic acid	-	-	254.49 ± 67.95	n.a	452.9 ± 96.0	249.3 ± 26.4	533.2 ± 36.7	n.a.	n.a	
**3a**	GA	GA	96.66 ± 33.94*	274.29 ± 9.42	236.33 ± 21.33*	248.5 ± 9.3	278.8 ± 10.8*	285.3 ± 5	273.16 ± 6.76	
**3b**	CA	CA	130.08 ± 37.8^	203.1 ± 47.7	242.8 ± 45.1^	193.6 ± 22^	251.4 ± 23.4^	280.7 ± 1.8	283.7 ± 6.3	
**7a**	PA	GA	60.89 ±10.86*	72.1 ±14.4	104.66 ± 6.51*	51.05±5.1*	54.96±2.12*	199.6±6.1	185.76 ± 15.2	
**7b**	PA	CA	45.66 ± 7.63^	61.2 ± 6.0	155.0 ± 88.3^	53.9 ± 2.97^	57.1 ± 2.5^	103.4 ± 11.1	111.9 ± 29.3	
**9a**	GA	-	323.5 ± 115.62	n.a.	522.96 ± 97.24	246.2 ± 9.9	526 ± 89.6	n.a.	n.a.	
**9b**	CA	-	381.58 ± 24.49	n.a	n.a	286.7 ± 35.1	551.9 ± 18.5	n.a.	n.a	
**10a**	GA	PA	38.21 ± 9.3*	49.54 ± 1.88	52.45 ± 5.9*	34.6 ± 1.6*	51.9 ± 6.7*	70.3 ± 8.6	65.8 ± 3.34	
**10b**	CA	PA	57.06 ± 6.37^	60.2 ± 6.4	208.7 ± 25.7^	52.4 ± 3.6^	57 ± 1.8^	232.9 ± 14.7	92.9 ± 15.3	
Cisplatin	-	-	1.3 ± 0.47	8.6 ± 0.7	8.1 ± 0.03	2.56±0.35	3.17±0.2	2.38±0.64	4.2 ± 1.1	
Doxorubicin	-	-	-	-	-	0.117±0.012	6.53±0.93	-	n.a.	

n.a.—no activity in concentration of 5, 25, 125, 625 μM. IC_50_ –compound concentration leading to 50% inhibition of cell proliferation. Data are presented as mean ± SD of 3–5 independent experiments.

*—results within column which are significantly different in comparison to geranic acid, p < 0.05.

^—results within column which are significantly different in comparison to citronellic acid, p < 0.05.

Using the obtained IC_50_ values, the resistance indexes (RI) were calculated by dividing the IC_50_ values of the compounds tested against the cells of drug resistant cell LOVO/DX line by respective values obtained against the cells of drug sensitive LoVo line. According to Harker et al. [26] three categories of the cells could be distinguished: (a) the cells are drug-sensitive—if the ratio approaches 0–2; (b) the cells are moderately drug-resistant—if the ratio ranges from 2 to 10; (c) the cells are markedly drug-resistant—if the ratio is higher than 10.

### Statistical analysis

Statistical analysis was performed using STATISTICA version 10 (StatSoft Inc., USA). Mann-Whitney U Test was used in the analysis and the results in Table 1 are given with statistical significance in comparison to geranic acid (*) or citronellic acid (^), p < 0.05.

## Results and Discussion

One of the most widely studied phospholipids is phosphatidylcholine (PC). Yamamoto proposed the transphosphatidylation of 1,2-dioleoyl-*sn*-glycero-3-phosphocholine (DOPC) with terpene alcohols (geraniol, nerol, perillyl alcohol, myrtenol, farnesol, geranylgeraniol, phytol [19,20] as the method for joining two biologically active groups of molecules. In this way the novel carrier-linked prodrugs—phospholipids containing terpenes in the polar part of PC, which showed antiproliferative effects on human leukaemia cells (HL-60) and prostate cancer (PC-3) cells were prepared [19,20]. Promising results obtained by Yamamoto inspired us to create a novel strategy for the synthesis of phosphatidylcholine enriched in biologically active terpenes. In our aproach we decided to leave polar part of phosphatidylcholine (PC) untouched, because choline is biologically active compound, which is a substrate for the synthesis of the neurotransmitter acetylcholine and directly affects many vital functions such as breathing, heart rate, memory processes. It also protects the liver cells, prevents accumulation of fat in hepative cells, lowers cholesterol levels, reduces the risk of atherosclerosis and heart diseases by the reduction of homocysteine level [27,28]. Instead, we used two monoterpene carboxylic acids (geranic and citronellic) as acyl donors and introduced them into apolar part of PC in order to extend their application field and ensure their efficient transport in the human body. Geranic acid is known as a strong inhibitor of the melanine synthesis and the most potent isoprenoid with antifungal activity towards *Fusarium graminearum* and *Colletotrichum graminicola* [29,30]. Its structural 2,3-dihydroanalogue named citronellic acid occurs in many essential oils and as the carrier of specific odor exhibiting antimicrobial activity is often used in the cosmetic industry as valuable ingredient of various preparations [31]. The biological activities of phospholipids depend on the composition of fatty acids in the *sn*-1 and *sn*-2 positions. From that reason three groups of terpene-modified phosphatidylcholines were synthesized. First group contained the same residue of geranic acid (GA) (**1a**) or cytronellic acid (CA) (**1b**) in the *sn*-1 and *sn*-2 positions. In second variant, GA or CA was attached to the *sn*-2 position whereas in *sn*-1 position saturated fatty acid (palmitic acid) was introduced as it is observed in natural phospholipids. Third group of phospholipids possessed geranic or cytronellic acid residue in *sn*-1 position and palmitic acid in *sn*-2 position.

**Fig 1:**
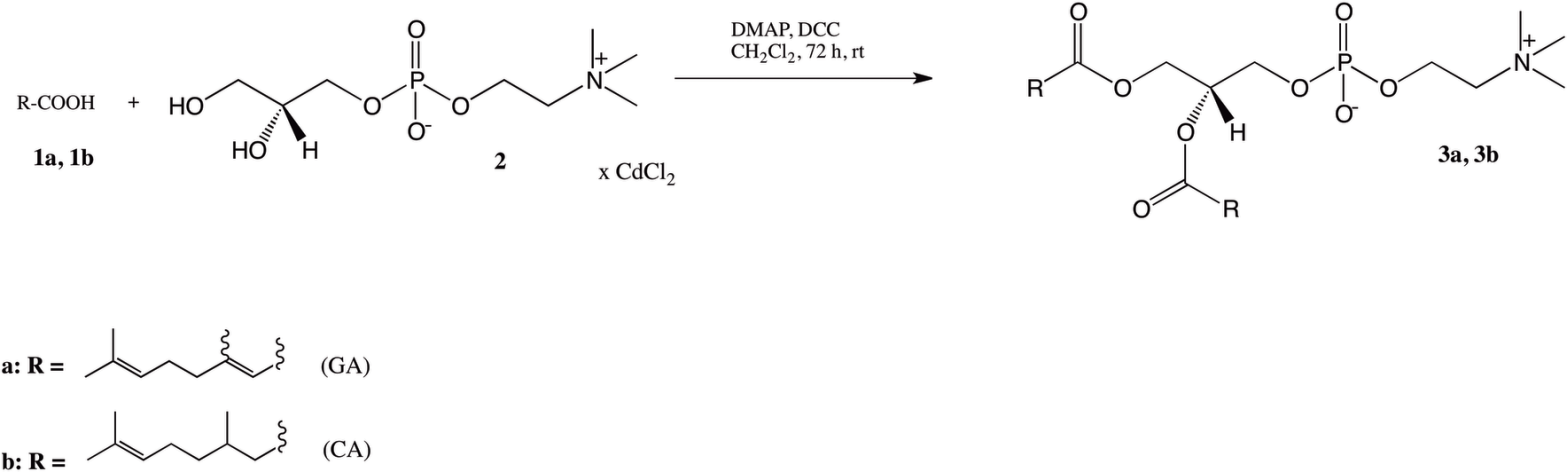
Synthesis of 1,2-diacyl-*sn*-glycero-3-phosphocholine containing the GA and CA residues (3a, 3b).

The first group of PC containing the same residue of geranic acid (**1a**) or citronellic acid (**1b**) in the *sn*-1 and *sn*-2 positions (**3a, 3b**) was obtained by known Steglich esterification method [32–34]. The cadmium complex of *sn*-glycero-3-phosphocholine (GPC×CdCl_2_) (**2**) was acylated with terpene acid (TA) **1a** and **1b** in the presence of 4-(*N*,*N*-dimethylamino)pyridine (DMAP) and a coupling agent *N*,*N’*-dicyclohexylcarbodiimide (DCC). All the reagents and solvent (dichloromethane) were freshly dried just before reaction to avoid the hydrolysis of DCC to dicyclohexylurea. In described condition using the molar ratio 1: 4: 2: 4.2 of GPC/TA/DMAP/DCC, 1,2-digeranoyl-*sn*-glycero-3-phosphocholine (**3a**) and 1,2-dicitroneloyl-*sn*-glycero-3-phosphocholine (**3b**) were obtained in 82% and 86% yields respectively after 72 hours of reaction (Fig 1).

The structures of products **3a** and **3b** were characterized based on comprehensive spectroscopic data interpretation. In the ^1^H NMR spectrum one can see signals from the incorporated isoprenoids. Signals from olefinic protons of terpene acids in the range δ 4.84–5.43 and protons from three methyl groups in δ 1.38–1.45 were observed. The carbon atoms of double bond systems present in terpenes acid residues gave signals in ^13^C NMR spectrum in the range 114.49–132.13 ppm for **3a** and in the range 123.64–131.09 ppm for **3b**. Signals in the ^31^P NMR spectrum at -0.63 ppm for **3a** and -0.79 ppm for **3b** proved that the phosphatidyl parts of molecules have been retained. Detailed assignments are given in the supplementary material (S1–S40 Figs).

**Fig 2:**
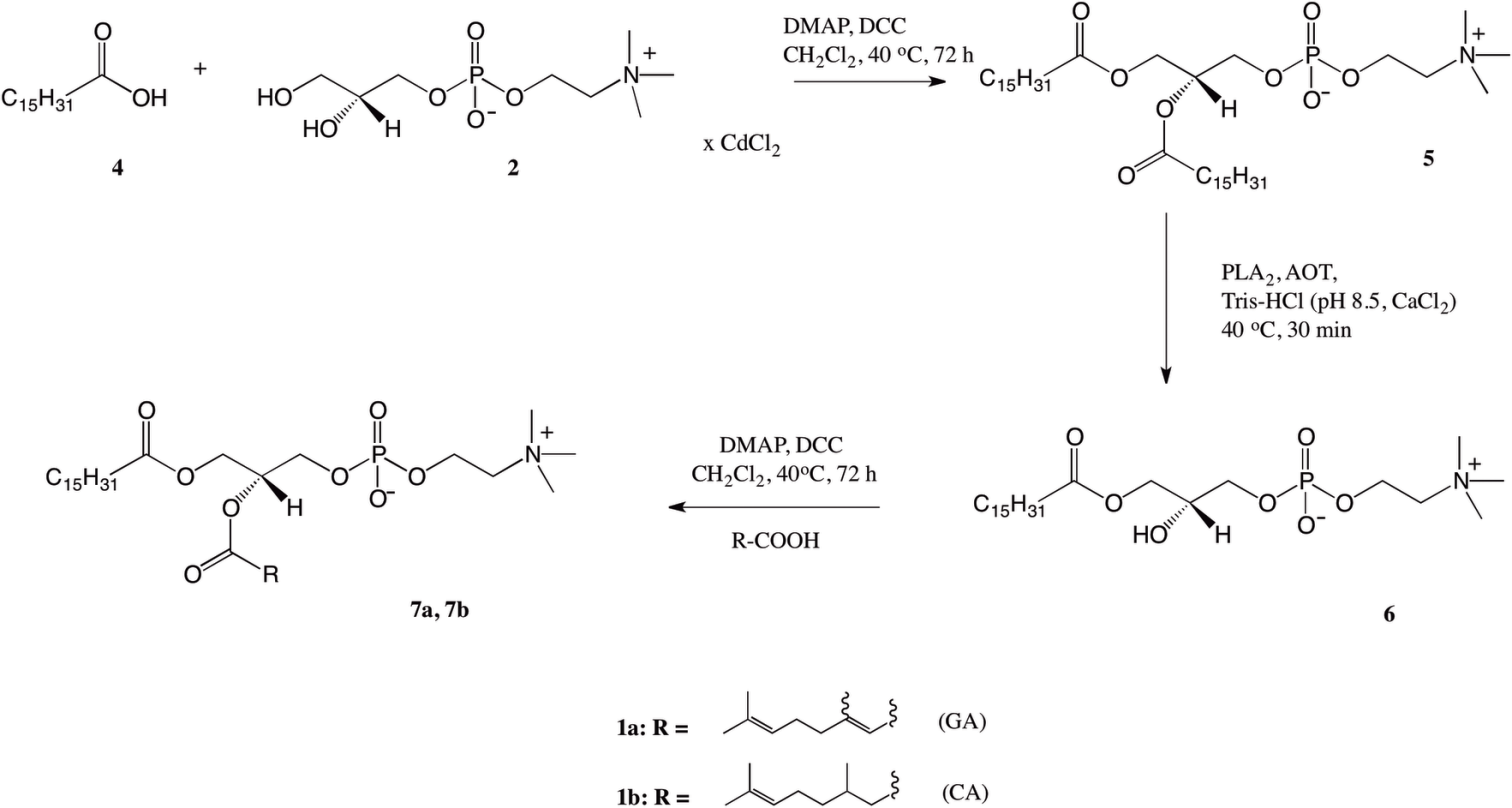
Synthesis of 1-palmitoyl-2-isoprenoyl-*sn*-glycero-3-phosphocholine (7a, 7b).

The synthesis of asymmetrically substituted phospholipids containing terpene acids (TA) in the *sn*-2 position and palmitic acid in *sn*-1 position was started by acetylation of cadmium complex of GPC with palmitic acid (PA) (Fig 2). Then 1,2-dipalmitoyl-*sn*-3-glycero-phosphocholine was converted to the 2-lysophospholipid (**6**) by treatment with phospholipase A_2_. Subsequent reacetylation of 2-OH group of known 1-palmitoyl LPC by isoprenoid acids yielded the mixed diacyl phosphatidylcholines (**7a, 7b**) [35].

**Fig 3:**
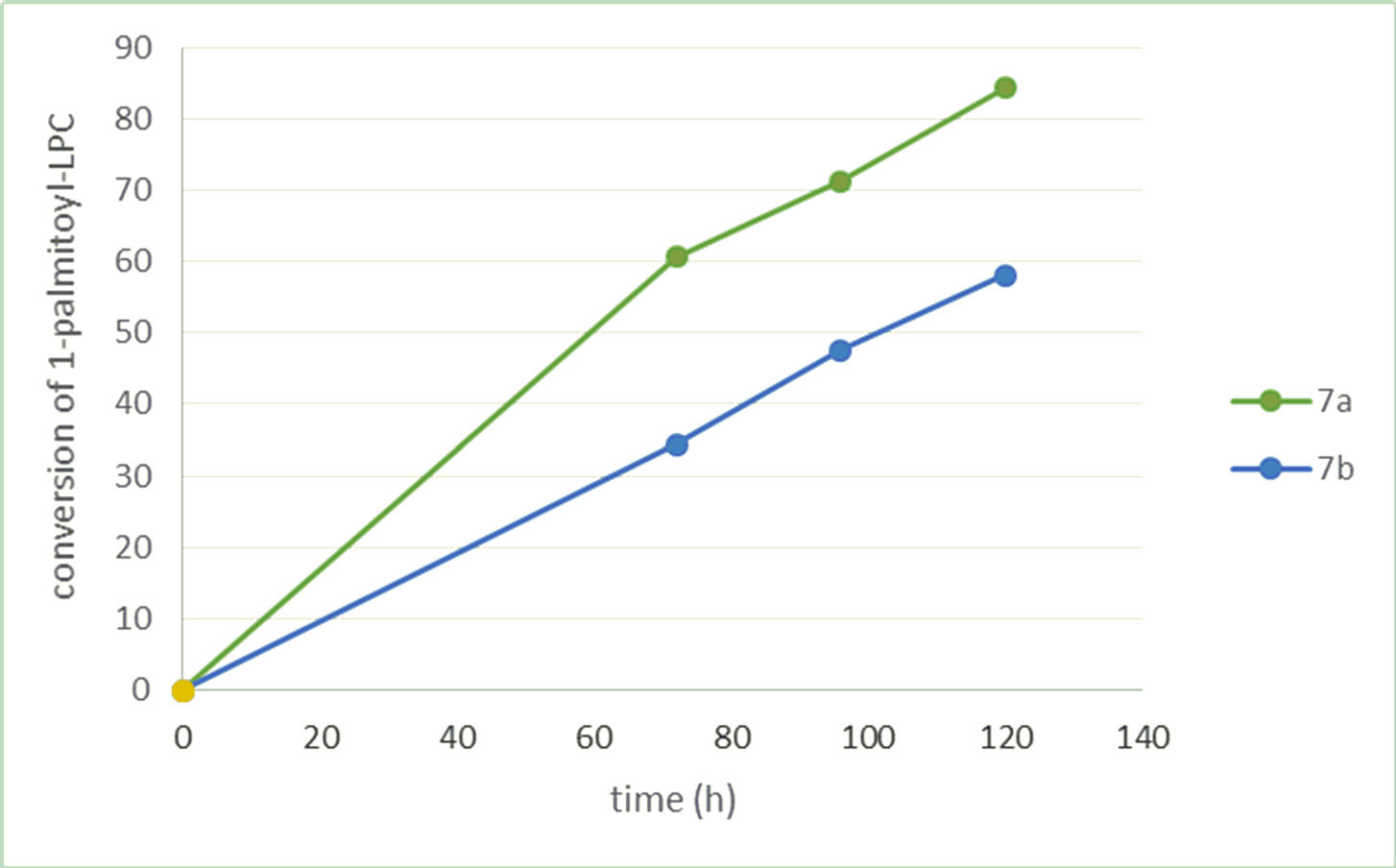
The time-course of esterification of 1-palmitoyl-2-hydroxy-*sn*-glycero-3-phosphocholine by isoprenoid acids.

The time-course of esterification of 1-palmitoyl-2-hydroxy-*sn*-glycero-3-phosphocholine by isoprenoid acids proceeded very slowly (Fig 3). We prolonged the time of reaction but still the isolated yields of asymmetrically substitued phosphatidylocholines were lower than in the case of symmetrically substituted products. The progress of the reaction was monitored by HPLC and showed that even after 120 hours the content of structured PC (**7a**, **7b**) in the reaction mixture was on the level of 58–84%. We decided to use then the higher molar excess of DCC and DMAP in relation of 1-palmitoyl-2-lysophospholipids and isoprenoid acids and increase temperature of reaction to 40°C which resulted the full conversion of substrate after 72 h of the reaction.

The structures of obtained products **7a** and **7b** were established based on their spectral data. The 1-palmitoyl-2-geranoyl-*sn*-glycero-3-phosphocholine (**7a**) was synthesized earlier by Biodrowska in 27% yield but in our investigations using higher ratio of DCC and DMAP resulted in significantly higher yield (59%) [36]. The 1-pamitoyl-2-citroneloyl-*sn*-glycero-3-phosphocholine (**7b)** has not been reported in the literature and was obtained in 62% yield.

**Fig 4:**
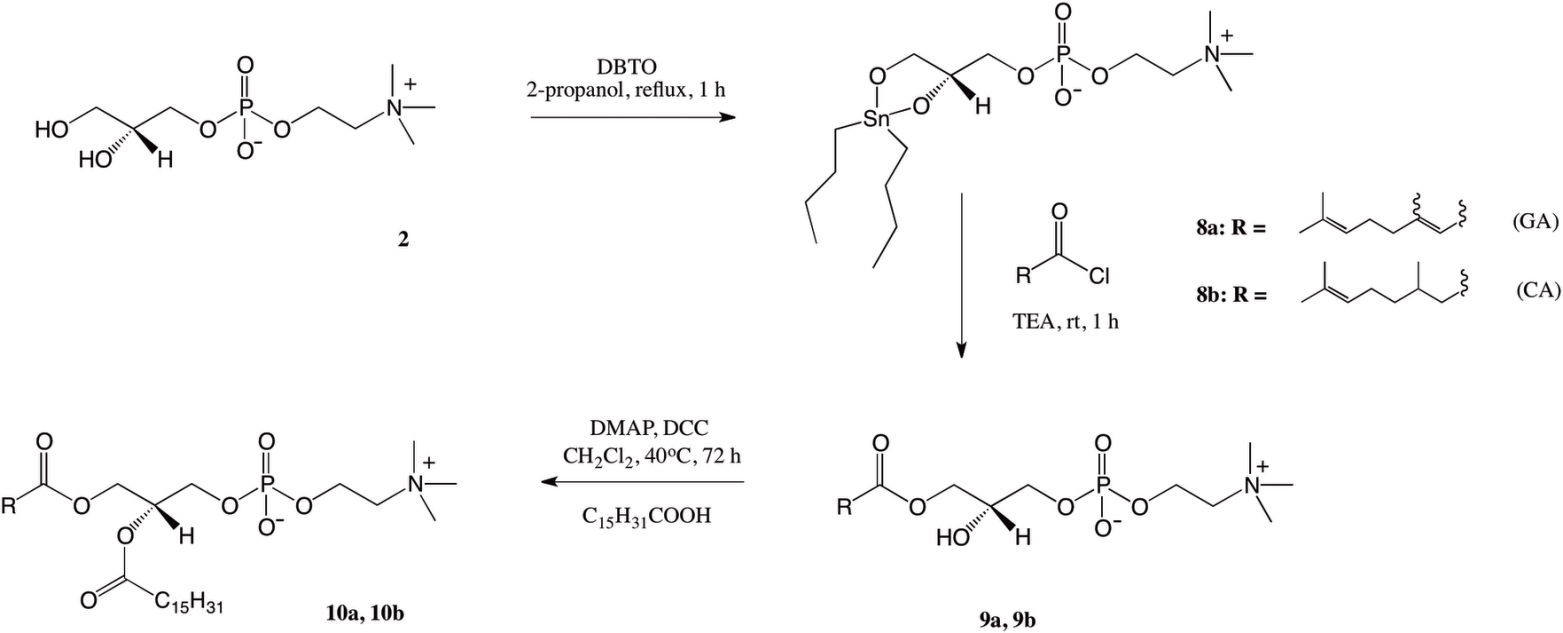
Synthesis of 1-isoprenoyl-2-palmitoyl-*sn*-glycero-3-phosphocholine (10a, 10b).

The synthesis of 1-isoprenoyl-2-palmitoyl-*sn*-glycero-3-phosphocholines is a task requiring regioselective incorporation of different acyl donors in two positions of the GPC backbone. The literature presents several useful methods for this type of mixed-chain phosphatidylcholines synthesis [29,24,37]. In all procedures the key step is production of 1-acyl-2-lyso-*sn*-glycero-3-phosphocholine. Initially, we applied chemoenzymatic strategy similar to that applied in the synthesis of compounds **7a** and **7b**. It was based on the hydrolysis of 1,2-diacyl-*sn*-glycero-3-phosphocholines **(3a, 3b)** obtained previously (Scheme 1) and subsequent reesterification of 1-acyl LPC with palmitic acid (PA) using DCC and DMAP. Unfortunately the yields of hydrolysis of terpene-PLs carried out with the phospholipase A_2_ (PLA_2_) were very low and we decided to use tin-mediated mono-functionalization of GPC described by D’Arrigo [35]. As it is presented in the Fig 4, in this process, GPC was first transformed into cyclic stannylene ketal by treatment with DBTO and then selectively acylated with isoprenoyl chlorides. Geranoyl and citroneloyl chlorides were obtained *in situ* using the procedure described by Mattson et al. [38]. We used the quantities of reagents suggested by D’Arrigo et al. [35], for the synthesis of 1-acyl-LPCs. Crude products were purified by a silica gel column chromatography to afford two new 1-isoprenoyl-2-hydroxy-*sn*-glycero-3-phosphocholines (**9a, 9b**) with 75% and 87% yields respectively. The last step was acylation of **9a** and **9b** with palmitic acid (PA) in the presence of DCC and DMAP, affording two new products **10a** and **10b** in 67 and 70% yields respectively. The structures of the final products were confirmed by their spectroscopic data.

All synthesized products (**3a**, **3b**, **7a**, **7b**, **9a**, **9b**, **10a**, **10b**) were obtained as the mixture of diastereoisomers. In the case of phospholipids containing geranic acid (**3a**, **7a**, **9a**, **10a**) it was the results of composition of *cis*/*trans* (23:67) isomers of starting isoprenoid. In the case of phosphatidylcholines modified with citronellic acid (**3b**, **7b**, **9b**, **10b**) the formation of two diastereoisomers (50:50) was caused by introduction of racemic citronellic acid into optically pure *sn*-glycero-3-phosphocholine (GPC).

### Biological studies

All terpene–modified PCs (**1a**, **1b**, **3a**, **3b**, **7a**, **7b**, **9a**, **9b**, **10a**, **10b**) were tested for their antiproliferative activity towards human cancer cell lines, such as MV4-11 (human leukaemia), A549 (lung cancer), MCF-7 (breast cancer), HepG2 (liver cancer), LoVo (colon cancer) and also against doxorubicin-resistant colon cancer LoVo/DX (P-gp-dependent, MRP-, LRP-dependent multidrug resistance). The experiments of cytotoxicity were also performed towards BALB/3T3 (normal mice fibroblasts). The data for the *in vitro* anticancer activity (Table 1) were expressed as the IC_50_ –concentration of the compound (in μM) that inhibits proliferation of cells by 50% compared to the untreated control cells. The results were compared with the activity of free terpene acids (TA). Cisplatin and doxorubicin hydrochloride (for LoVo and LoVo/Dx cells) were used as a positive control.

The aim of this study was to determine whether a joining the terpenes with PC has impact on their biological activity. Increasing the lipophilicity of active compounds was expected to raise their cytotoxicity. We observed that almost all synthesized compounds were cytotoxic against tested cancer cell lines. The exception is compound **9a** that is not active towards cells of A549 and HepG2 lines and **9b**, which inhibits only the proliferation of leukaemia and colon cancer LoVo cells but in high concentration on the level 381.58 μM and 286.7 μM, respectively. Upon careful evaluation of data it can be concluded that cytotoxic activity of terpene-modified PCs is depended on the position of terpene moiety in the backbone of PC. Symmetrically substituted phospholipids (**3a**, **3b**) containing terpene acids (TA) in the *sn*-1 and *sn*-2 position possessed the antiproliferative activity 2-3-fold higher than free acids. These compounds stronger than corresponding acids inhibit the proliferation of cancer cells. Free terpene acids (**1a**, **1b**) did not showed any reduction of cells growth of A549, HepG2 even at 625 μM. The highest activity was observed for mixed-chain PC (**7a**, **7b**, **10a**, **10b**). Their IC_50_ values were more than 9-fold lower than those determined for free terpene acids **1a**, **1b** towards leukaemia, LoVo, LOVO/DX and breast cell lines. For cells of lung cancer and HepG2 lines the activity was exhibited only after introduction of terpene acids into PC. Among eight tested terpene-PCs compound **10a** showed the strongest antiproliferative effect towards all tested cell lines. Very high antiproliferative activity was demonstrated against leukaemia and colon cancer LoVo cells (IC_50_ 38.21 and 34.6 μM, respectively). During the experiments we observed also that tested compounds exhibit activity against normal mice fibroblasts (BALB/3T3). However it is worth to notice that cytotoxic activity of compounds **7a**, **7b**, **10a** and **10b** against BALB/3T3 cells was in most cases lower in comparison to cytotoxicity exhibited against cancer cell lines, which is very promising in contest of potential application of tested compounds as the anticancer drugs. Generally, activity of terpene-modified PC increased in the following sequence: 1-isoprenoyl-LPC < diisoprenoyl-PC < monoisoprenoyl-PC.

We calculated also the resistance indexes (RI) by dividing the IC_50_ values of the compounds tested against the cells of drug resistant cell LoVo/DX by respective values obtained against the drug sensitive LoVo cell line (Table 2). All compounds were able to overcome the barrier of P-gp-dependent resistance with exception of two compounds: free terpene acids **1b** and **9a**, which revealed moderated ability to overcome drug resistance. Compounds **7a**, **7b** and **10b** have the highest ability to overcome the barrier of resistance (RI = 1.08, 1.06 and 1.09).

**Table 2:** Resistance index (RI) values of terpene-phospholipids.

Compounds	Acyl residue		
	*sn*-1	*sn*-2	RI
**1a** geranic acid	-	-	1.87
**1b** citronellic acid	-	-	2.14
**3a**	GA	GA	1.12
**3b**	CA	CA	1.3
**7a**	PA	GA	1.08
**7b**	PA	CA	1.06
**9a**	GA	-	2.14
**9b**	CA	-	1.93
**10a**	GA	PA	1.5
**10b**	CA	PA	1.09
**DOX**	-	-	55.81

DOX–doxorubicin

**RI** was calculated according to the formula RI = (IC_50_ estimated against resistant cell line)/(IC_50_ estimated against non-resistant cell line); values range: 0<RI<2-indicate that the tested compound is able to overcome drug resistance; 2<RI<10 –defines the moderate ability of the compound to overcome drug resistance; RI>10 –defines no influence on the drug resistance phenomenon.

In conclusion, all novel phospholipids containing the isoprenoid residue of geranic and citronellic acid in the non-polar part of phosphatidylcholine were synthesized in good yields (59–87%). It is the first report on the evaluation of anticancer activity of geranic and citronellic acid in the free form and after incorporation into PC. The results of cytotoxic studies confirmed that phospholipid modifications with geranic and citronellic acids enhanced their biological activity. It was indicated by the lower active doses of terpene-PCs comparison to doses of free terpene acids. The low activity observed for lysoderivatives may be a result of significantly increased polarity of those compounds comparing to 1,2-diacylphosphatidylcholines. We found that there is structure-activity relationship among the tested terpene-modified PCs but it is also important to determine how the structure of isoprenoid residue (the chain length, number of double bond *etc*.) influences on the activity of whole molecule. These studies are in progress. The aim of further work is also to elucidate the mechanism of the cytotoxicity terpene-modified PCs.

## Supporting Information

S1 Fig ^1^H NMR spectrum of 3a. (DOCX)

S2 Fig ^13^C NMR spectrum of 3a. (DOCX)

S3 Fig ^31^P NMR spectrum of 3a. (DOCX)

S4 Fig ^1^H – ^1^H COSY spectrum of 3a. (DOCX)

S5 Fig HSQC spectrum of 3a. (DOCX)

S6 Fig ^1^H NMR spectrum of 3b. (DOCX)

S7 Fig ^13^C NMR spectrum of 3b. (DOCX)

S8 Fig ^31^P NMR spectrum of 3b. (DOCX)

S9 Fig ^1^H – ^1^H COSY spectrum of 3b. (DOCX)

S10 Fig HSQC spectrum of 3b. (DOCX)

S11 Fig ^1^H NMR spectrum of 7a. (DOCX)

S12 Fig ^13^C NMR spectrum of 7a. (DOCX)

S13 Fig ^31^P NMR spectrum of 7a. (DOCX)

S14 Fig ^1^H – ^1^H COSY spectrum of 7a. (DOCX)

S15 Fig HSQC spectrum of 7a. (DOCX)

S16 Fig ^1^H NMR spectrum of 7b. (DOCX)

S17 Fig ^13^C NMR spectrum of 7b. (DOCX)

S18 Fig ^31^P NMR spectrum of 7b. (DOCX)

S19 Fig ^1^H – ^1^H COSY spectrum of 7b. (DOCX)

S20 Fig HSQC spectrum of 7b. (DOCX)

S21 Fig ^1^H NMR spectrum of 9a (DOCX)

S22 Fig ^13^C NMR spectrum of 9a. (DOCX)

S23 Fig ^31^P NMR spectrum of 9a. (DOCX)

S24 Fig ^1^H – ^1^H COSY spectrum of 9a. (DOCX)

S25 Fig HSQC spectrum of 9a. (DOCX)

S26 Fig ^1^H NMR spectrum of 9b. (DOCX)

S27 Fig ^13^C NMR spectrum of 9b. (DOCX)

S28 Fig ^31^P NMR spectrum of 9b. (DOCX)

S29 Fig ^1^H – ^1^H COSY spectrum of 9b. (DOCX)

S30 Fig HSQC spectrum of 9b. (DOCX)

S31 Fig ^1^H NMR spectrum of 10a. (DOCX)

S32 Fig ^13^C NMR spectrum of 10a. (DOCX)

S33 Fig ^31^P NMR spectrum of 10a. (DOCX)

S34 Fig ^1^H – ^1^H COSY spectrum of 10a. (DOCX)

S35 Fig HSQC spectrum of 10a. (DOCX)

S36 Fig ^1^H NMR spectrum of 10b. (DOCX)

S37 Fig ^13^C NMR spectrum of 10b. (DOCX)

S38 Fig ^31^P NMR spectrum of 10b. (DOCX)

S39 Fig ^1^H – ^1^H COSY spectrum of 10b. (DOCX)

S40 Fig HSQC spectrum of 10b. (DOCX)
