# Supplementary figures and images for: Synthesis and Biological Evaluation of Novel Phosphatidylcholine Analogues Containing Monoterpene Acids as Potent Antiproliferative Agents
Source: PLoS One. 2016 Jun 16;11(6):e0157278. doi: 10.1371/journal.pone.0157278 (PMC4911001; doi:10.1371/journal.pone.0157278)

## S1 Fig. 1H NMR spectrum of 3a


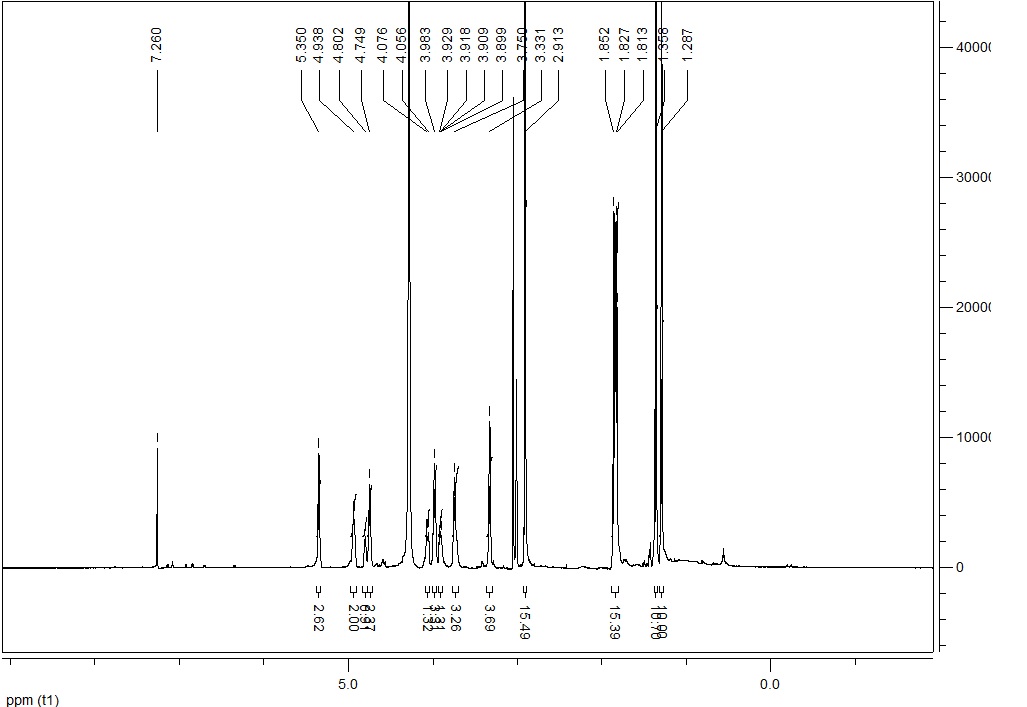

Supplement: S1 Fig — (DOCX) [file pone.0157278.s001.docx]

## S2 Fig. 13C NMR spectrum of 3a


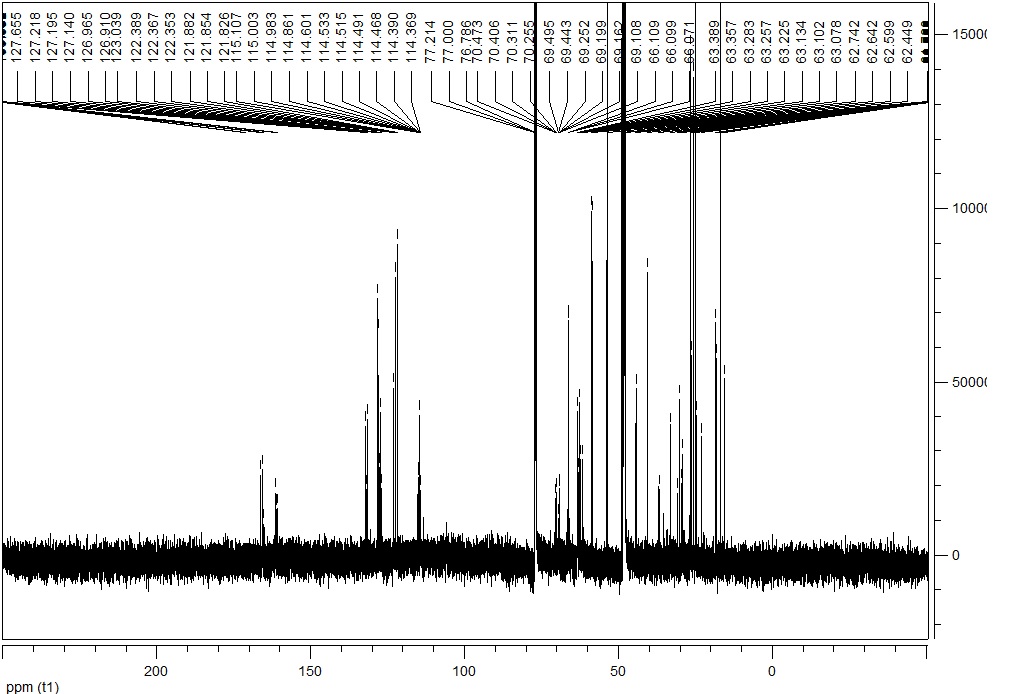

Supplement: S2 Fig — (DOCX) [file pone.0157278.s002.docx]

## S3 Fig. 31P NMR spectrum of 3a


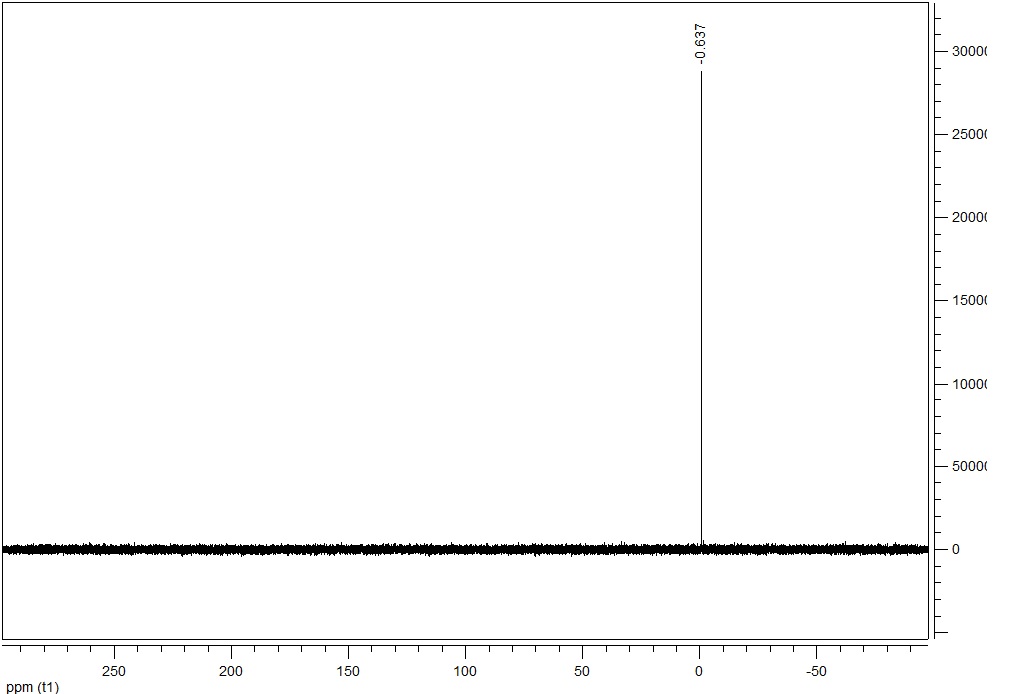

Supplement: S3 Fig — (DOCX) [file pone.0157278.s003.docx]

## S6 Fig. 1H NMR spectrum of 3b


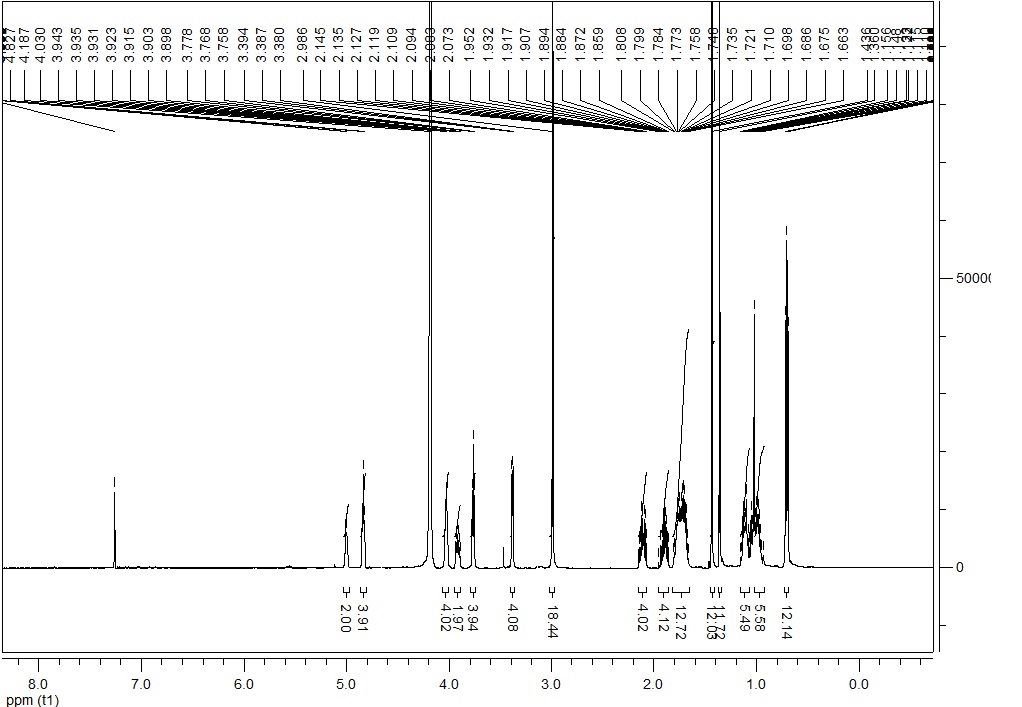

Supplement: S6 Fig — (DOCX) [file pone.0157278.s006.docx]

## S11 Fig. 1H NMR spectrum of 7a


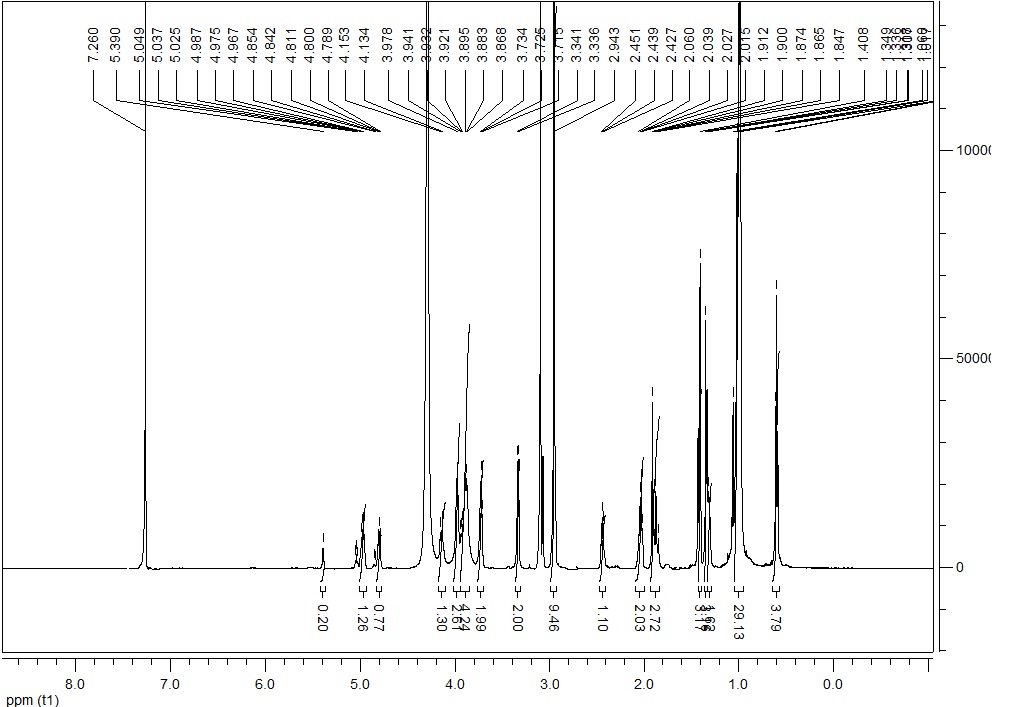

Supplement: S11 Fig — (DOCX) [file pone.0157278.s011.docx]

## S12 Fig. 13C NMR spectrum of 7a


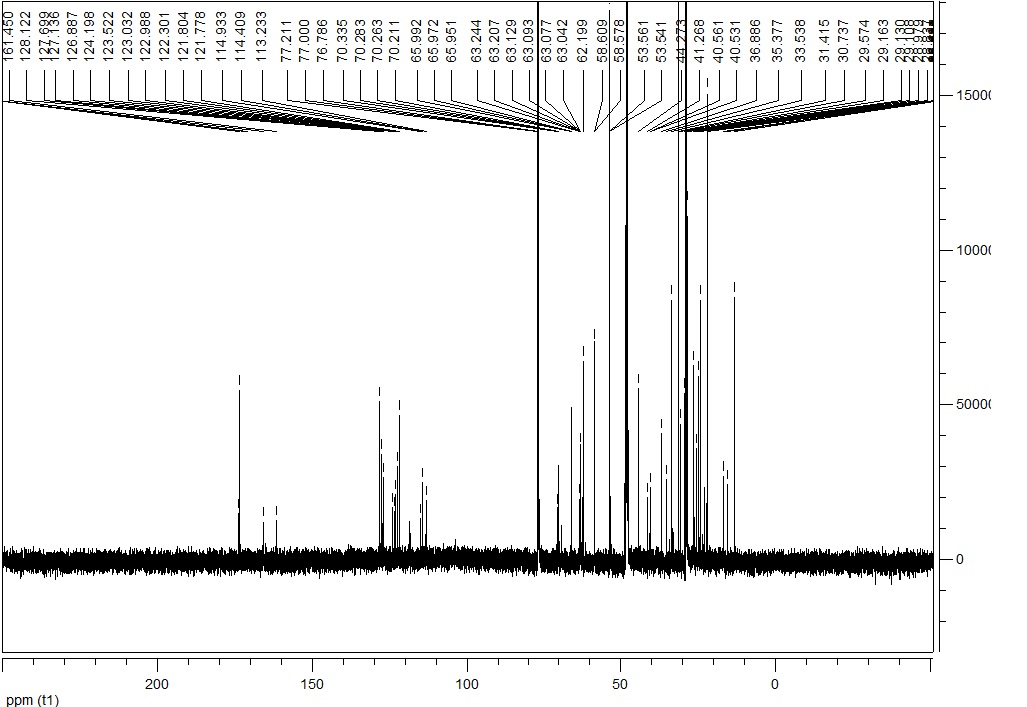

Supplement: S12 Fig — (DOCX) [file pone.0157278.s012.docx]

## S13 Fig. 31PNMR spectrum of 7a


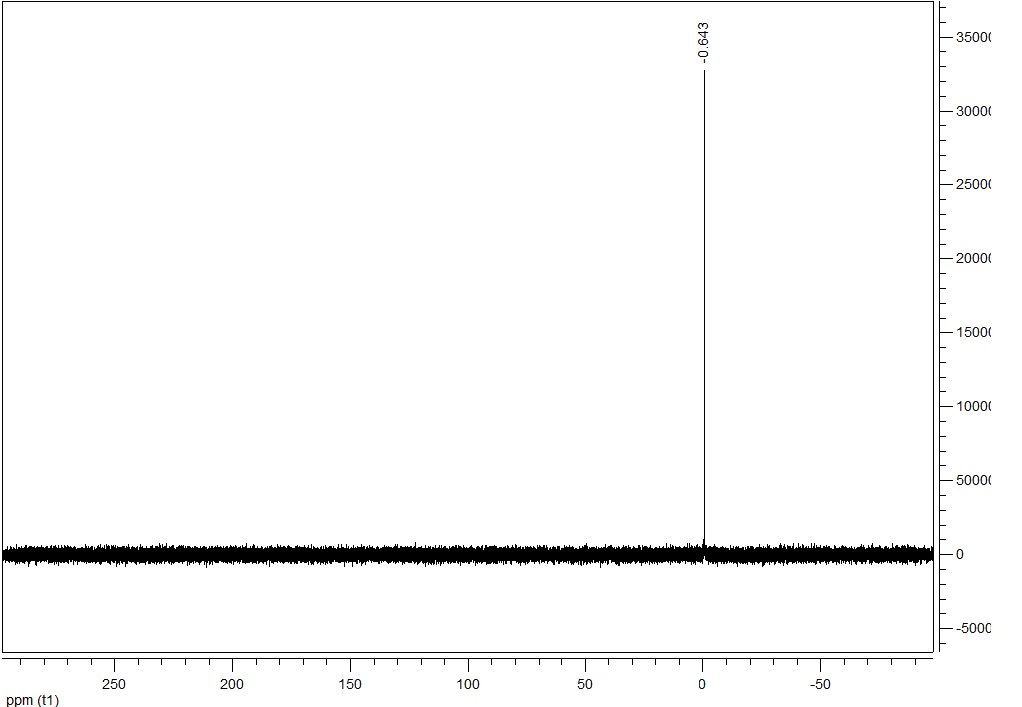

Supplement: S13 Fig — (DOCX) [file pone.0157278.s013.docx]

## S16 Fig. 1H NMR spectrum of 7b


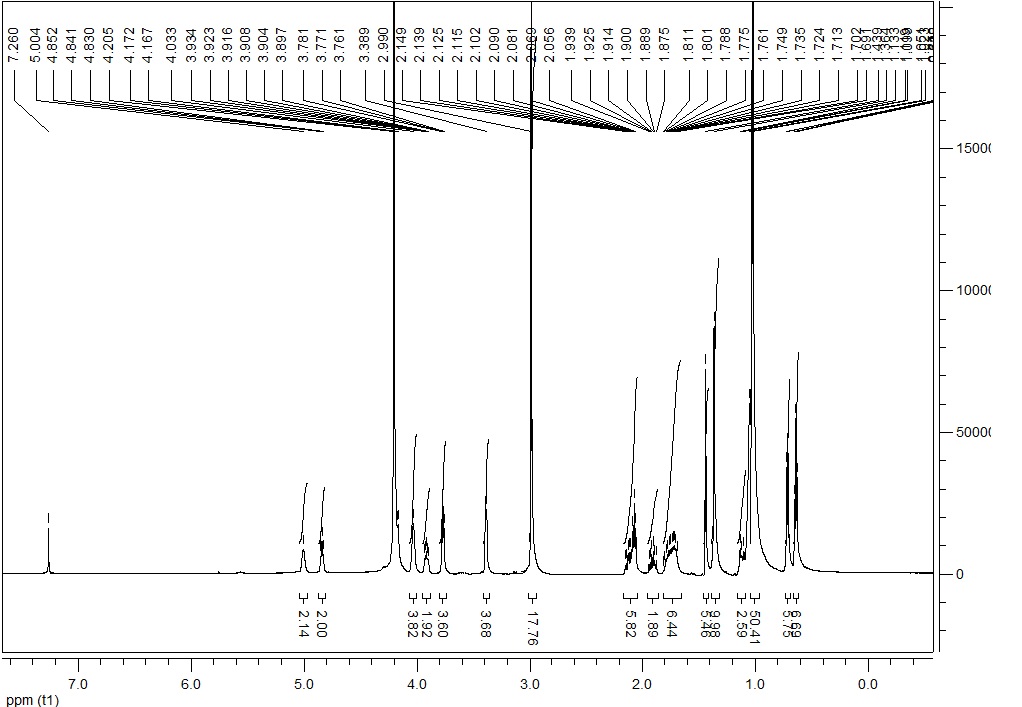

Supplement: S16 Fig — (DOCX) [file pone.0157278.s016.docx]

## S18 Fig. 31PNMR spectrum of 7b


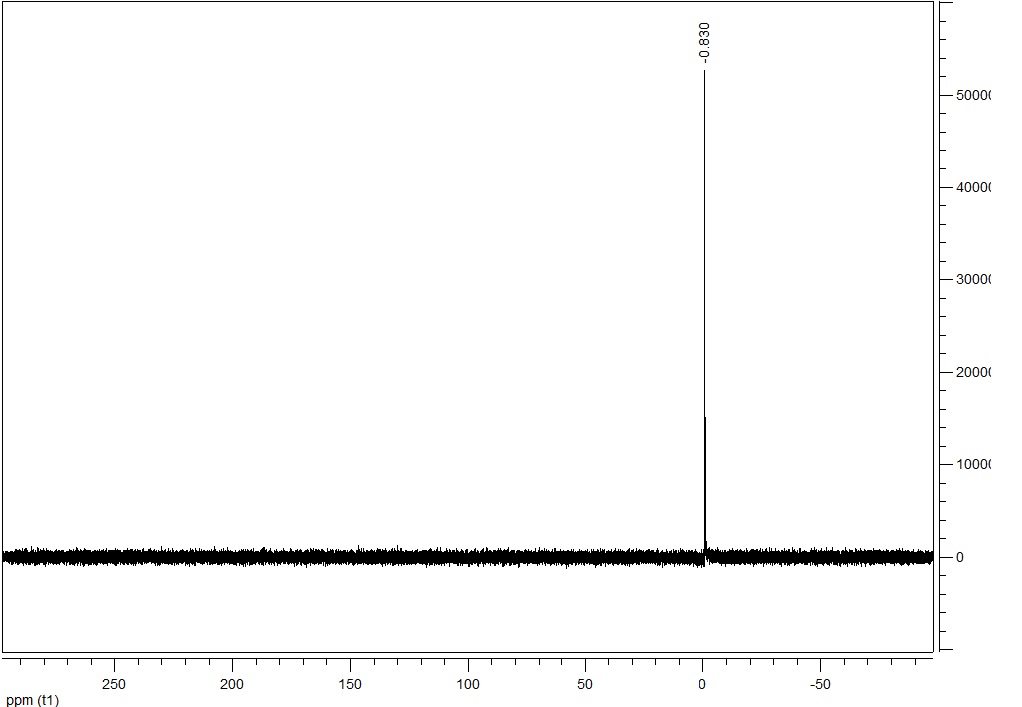

Supplement: S18 Fig — (DOCX) [file pone.0157278.s018.docx]

## S21 Fig. 1H NMR spectrum of 9a


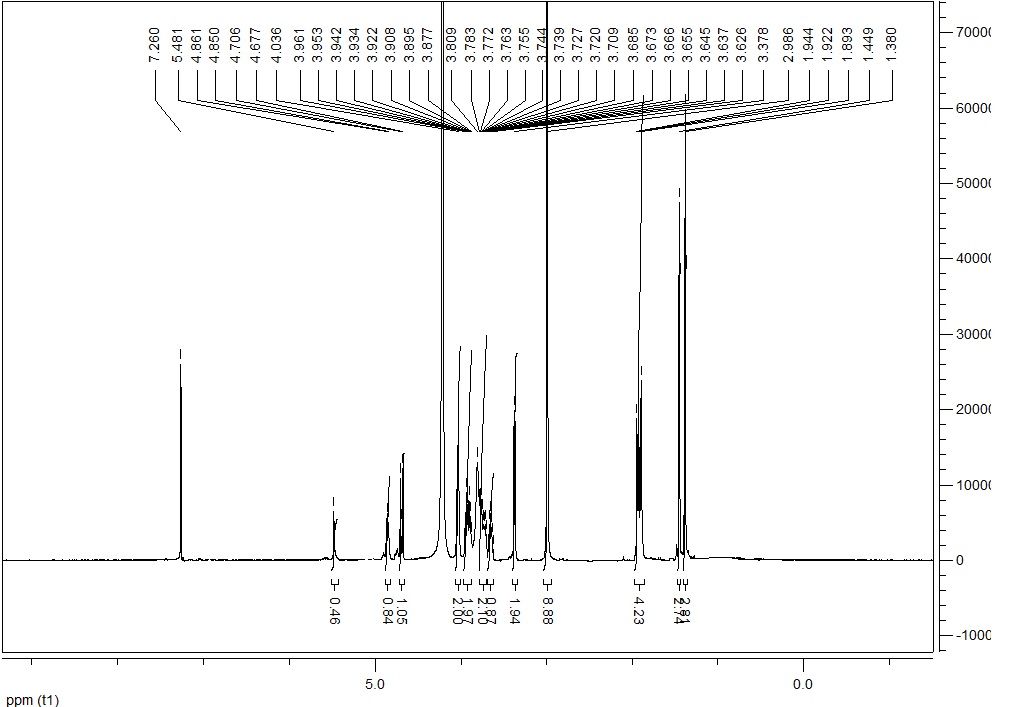

Supplement: S21 Fig — (DOCX) [file pone.0157278.s021.docx]

## S22 Fig. 13C NMR spectrum of 9a


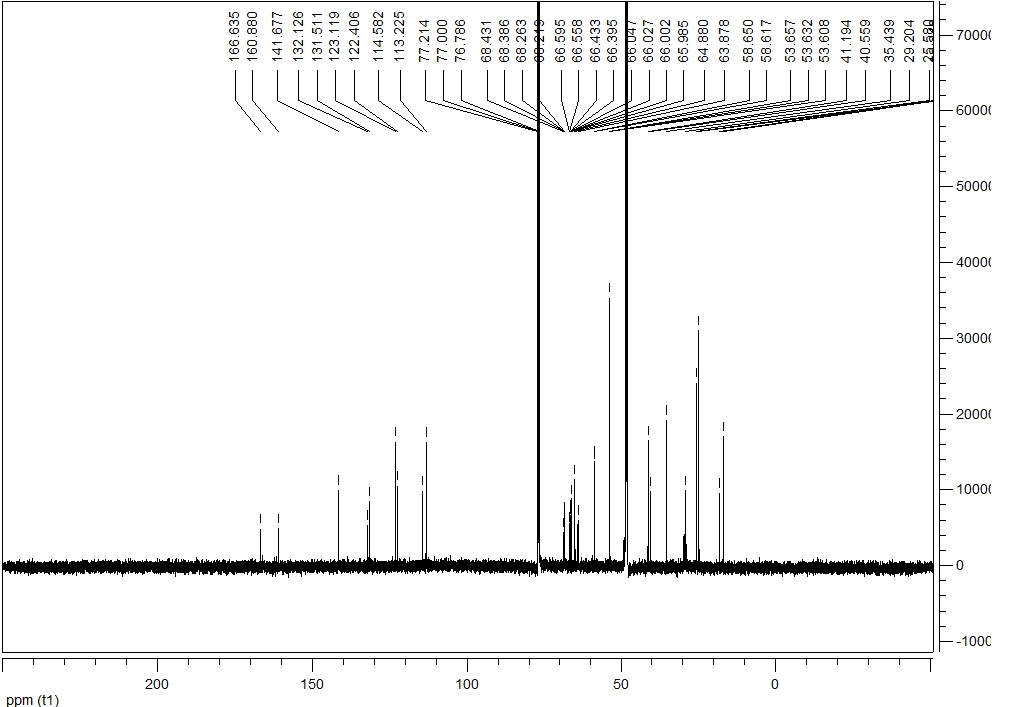

Supplement: S22 Fig — (DOCX) [file pone.0157278.s022.docx]

## S23 Fig. 31PNMR spectrum of 9a


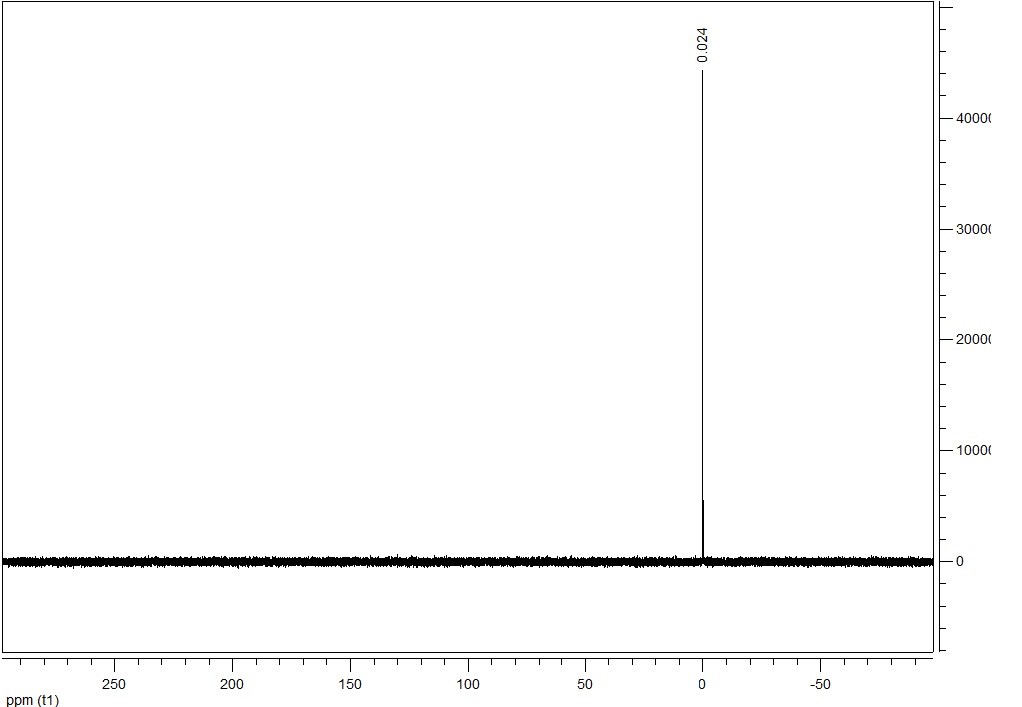

Supplement: S23 Fig — (DOCX) [file pone.0157278.s023.docx]

## S26 Fig. 1H NMR spectrum of 9b


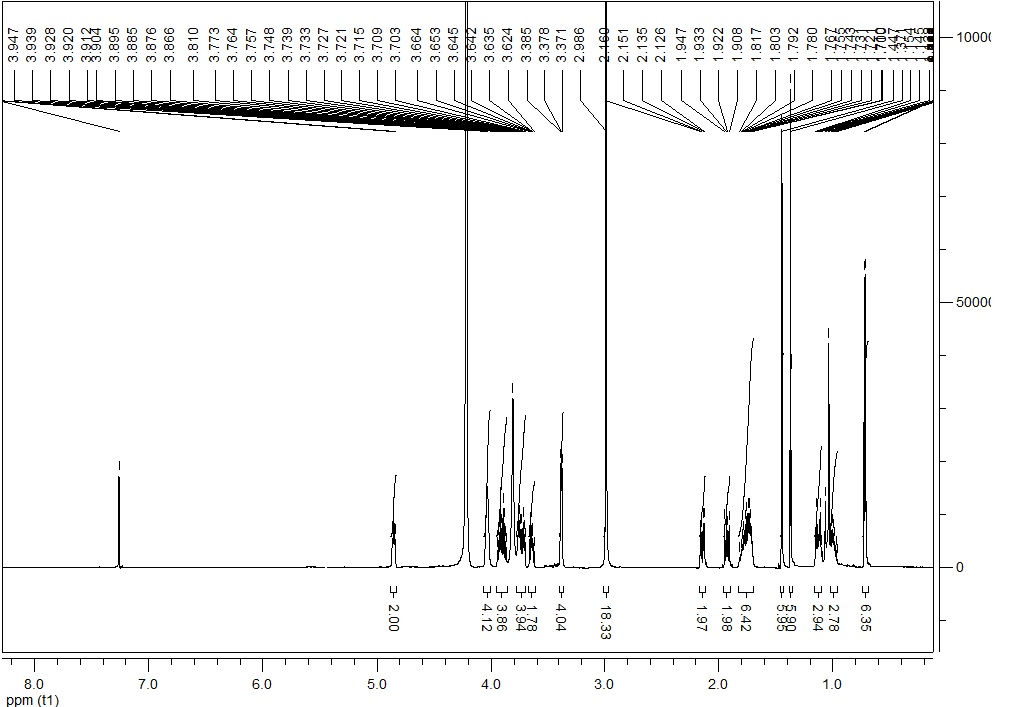

Supplement: S26 Fig — (DOCX) [file pone.0157278.s026.docx]

## S31 Fig. 1H NMR spectrum of 10a


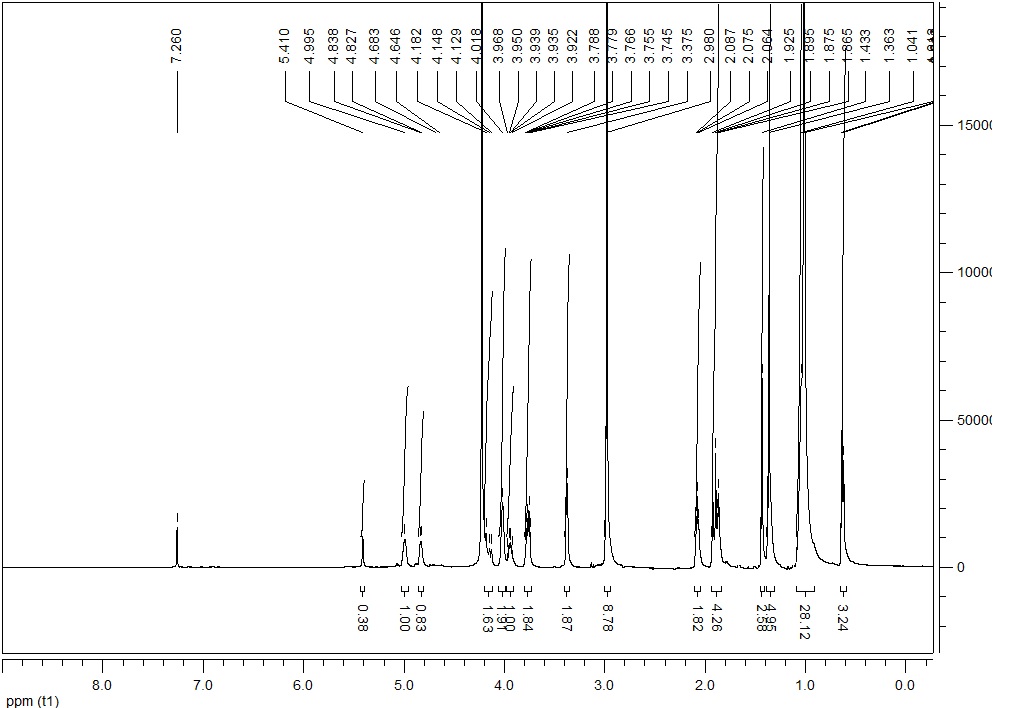

Supplement: S31 Fig — (DOCX) [file pone.0157278.s031.docx]

## S32 Fig. 13C NMR spectrum of 10a


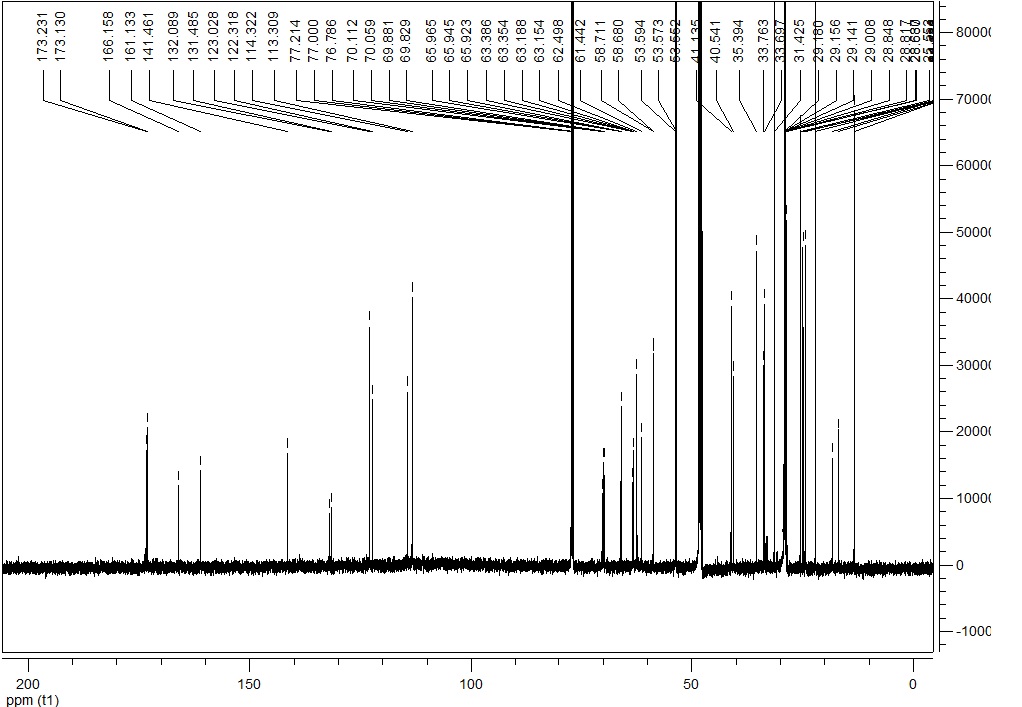

Supplement: S32 Fig — (DOCX) [file pone.0157278.s032.docx]

## S36 Fig. 1H NMR spectrum of 10b


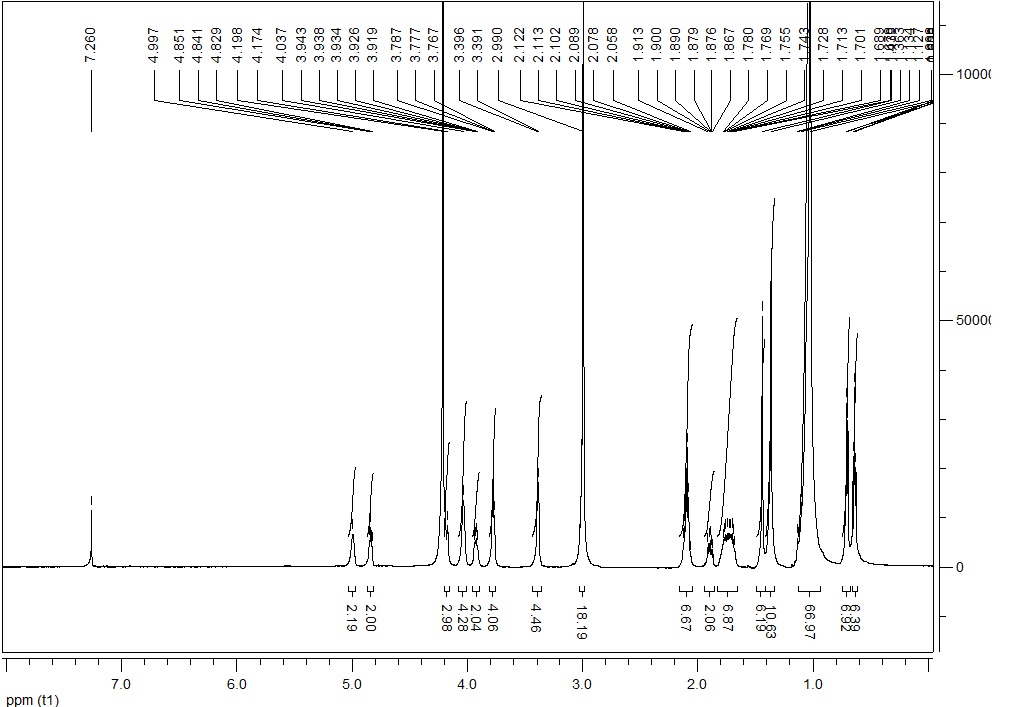

Supplement: S36 Fig — (DOCX) [file pone.0157278.s036.docx]
