# Supplementary material for: Synthesis and Biological Evaluation of Novel Phosphatidylcholine Analogues Containing Monoterpene Acids as Potent Antiproliferative Agents
Source: PLoS One. 2016 Jun 16;11(6):e0157278. doi: 10.1371/journal.pone.0157278 (PMC4911001; doi:10.1371/journal.pone.0157278)
Supplement: S15 Fig — (DOCX) [file pone.0157278.s015.docx]

## S15 Fig. HSQC spectrum of 7a
